# Do high-level badminton players show broad executive-function advantages? Evidence from a multi-task assessment

**DOI:** 10.3389/fpsyg.2026.1960617

**Published:** 2026-10-27

**Authors:** Shaohan Sun, Zhao Zhang

**Affiliations:** 1 College of Physical Education and Health, East China Normal University, Shanghai, China; 2 School of Sports Training, Chengdu Sport University, Chengdu, China

**Keywords:** badminton, badminton performance level, executive function, processing speed, response time

## Abstract

**Background:**

Cognitive differences across badminton performance levels may be task-specific rather than reflect broad executive-function advantages.

**Methods:**

Ninety-six participants (33 high-level players, 31 recreational players and 32 non-badminton participants) completed word-color matching, task switching, 1-back and Raven testing. Analyses adjusted for sex, age and Raven score. Task-level MANCOVAs used Pillai’s trace, with HC3-adjusted RT models and binomial mixed models for follow-up comparisons. Additional models distinguished 1-back timeouts from accuracy among answered trials.

**Results:**

Overall group effects were significant for word-color matching (*p* < 0.001) and 1-back (*p* = 0.011), but not task switching (*p* = 0.592). High-level players responded faster in both word-color conditions than both comparison groups. Condition, response hand and response frequency were confounded, preventing an isolated interpretation of the condition RT contrast. In 1-back, timeout rates were 40.45%, 36.92% and 48.21% in the high-level, recreational and non-badminton groups; both badminton groups had lower adjusted timeout rates than the non-badminton group in both conditions. Accuracy among answered trials showed no clear group differences, and correct-response RT did not differ significantly (*p* = 0.572). All-trial accuracy findings were sensitive to model specification.

**Conclusion:**

High-level players showed faster word-color matching, and both badminton groups had fewer timeouts in the deadline-constrained 1-back task. The findings indicate task-specific performance differences rather than a broad executive-function advantage.

## 1 Introduction

High-level sport depends on physical capacity and technical skill. It also draws on domain-specific and domain-general cognitive processes (Kalén et al., 2021). Identifying task-relevant cues and making decisions under distraction place demands on working memory and attentional control (Furley and Wood, 2016). Badminton is an open-skill sport characterized by high shuttle speed, rapidly changing trajectories, and frequent transitions between attack and defense; during play, athletes must extract visual information and adjust their actions under time pressure (Phomsoupha and Laffaye, 2015; Gu et al., 2019).

Executive functions are higher-order processes that support goal-directed behavior (Diamond, 2013). Inhibition, shifting, and updating are commonly described as related but distinguishable components (Miyake et al., 2000). Badminton-specific studies have reported more accurate landing-position anticipation and distinct P2/P300 responses in professional players (Jin et al., 2011), as well as shorter inhibition and change-response latencies in a change-signal task among high-level players (Chen et al., 2018). These findings link badminton expertise to anticipation and action-control tasks, but they do not establish a general advantage across executive-function components.

Evidence across sports is mixed. Executive-function scores have predicted competitive success in elite footballers (Vestberg et al., 2012), yet multi-task assessments show that expertise, sport type, sex, and score construction do not produce uniform effects (Vona et al., 2024). Open-skill athletes sometimes outperform closed-skill athletes on working-memory or cognitive-flexibility tasks, but prior participation in both sport types also matters (Koch and Krenn, 2021). Short bouts of badminton have altered performance under an incongruent word-color condition in some studies (Takahashi and Grove, 2019), and higher-level adolescent badminton players have shown better spatial working-memory performance (Wu et al., 2024). Systematic reviews nevertheless report substantial variation across tasks and samples (Lai et al., 2024). Recent latent-variable work in athletes also supports both common and component-specific aspects of executive functioning (Brimmell et al., 2025).

General cognitive ability is another source of variation. Fluid intelligence supports abstract reasoning and problem solving in novel situations (Cattell, 1963). It overlaps with executive functions at behavioral and neural levels without being identical to them (Santarnecchi et al., 2021). Relations between fluid intelligence, working-memory capacity, and specific executive processes depend on how each construct is decomposed (Unsworth et al., 2009; Ren et al., 2017). Sex-related differences are inconsistent across executive-function tasks (Grissom and Reyes, 2019), whereas age-related changes extend through adolescence and adulthood (Ferguson et al., 2021). Sex, age, and fluid intelligence were therefore treated as covariates.

Badminton research has largely focused on selected tasks, outcomes, or age groups. It remains unclear whether performance differences across badminton performance levels reflect inhibition, shifting, or updating, or instead arise from processing operations shared across task conditions. To address this question, we compared accuracy, reaction time, inverse efficiency, and component-specific condition contrasts across three executive-function task paradigms. By examining multiple executive-function tasks within the same sample and adjusting for sex, age, and fluid intelligence, the study aimed to distinguish task-level performance differences from component-specific executive-function advantages. Comparing performance levels within badminton also reduced the cross-sport heterogeneity inherent in comparisons between different sport categories.

On the basis of this evidence, three hypotheses were tested after adjustment for sex, age, and fluid intelligence. First (H1), the high-level group was expected to respond more quickly than the recreational and non-badminton groups in both word-color matching conditions without lower accuracy, and to show a smaller condition reaction-time difference. Second (H2), the high-level group was expected to show higher accuracy and shorter correct-response time under both switch and repeat conditions, together with a smaller switch cost. Third (H3), the high-level group was expected to show higher accuracy and shorter correct-response time on the 1-back task. This prediction is evaluated as deadline-constrained 1-back performance rather than as an isolated measure of updating ability. Because IES is calculated from reaction time and accuracy, it was treated as a derived supporting outcome rather than an independent test of these hypotheses. The hypotheses were formulated before the data were examined or analyzed. The predicted smaller word-color condition difference reflected an expectation of more efficient response selection under conflicting cues in high-level athletes. With fixed keys and unequal response frequencies, the condition-difference component of H1 cannot independently test a conflict effect in this implementation.

## 2 Materials and methods

### 2.1 Study design and setting

This was a cross-sectional, three-group observational study reported in accordance with the Strengthening the Reporting of Observational Studies in Epidemiology (STROBE) statement (von Elm et al., 2007). Participants in the recreational and non-badminton groups were recruited from sport-related degree programs, whereas the high-level group was recruited from the Chengdu Sport University badminton team. Data were collected at Chengdu Sport University between 31 October and 30 December 2025. Testing was conducted in a quiet sport psychology laboratory using a single Lenovo Xiaoxin Pro 16 laptop with its 16-inch built-in display set to 120 Hz and an external Logitech K251 wireless keyboard. No external monitor was used. All participants used the same equipment and settings. The viewing distance was approximately 60 cm. Participants positioned their left and right index fingers on the *V* and *N* keys, respectively. Handedness was recorded for each participant. All participants used *V* for same/match and *N* for different/mismatch.

All participants completed the tasks in a fixed order: word-color matching, task switching, 1-back, and Raven’s Standard Progressive Matrices. Inter-task intervals lasted approximately 3 min, allowing participants to rest while the experimenter switched programs and entered participant information.

### 2.2 Participants

Ninety-six participants aged 18–24 years in the final sample were enrolled in degree programs in sports training, physical education, and leisure sports. The sample comprised 63 men and 33 women. All participants reported normal or corrected-to-normal vision, no color-vision deficiency, and no history of neurological or psychiatric disease. The protocol was approved by the Ethics Committee of Chengdu Sport University (CDSU Ethics [2025] 157; approved 29 September 2025), and all participants provided written informed consent. Of 127 individuals approached, 30 declined participation and none were excluded at eligibility screening. Ninety-seven began testing. One woman in the non-badminton group withdrew after completing the word-color task because of an unexpected scheduling conflict. Her data were removed and were not included in any analysis, leaving 96 participants.

Sex was recorded as reported by participants, coded as 1 for men and 2 for women, and included as a covariate. The study was not designed or powered for sex-stratified inference.

Participants were classified into three naturally occurring groups. The high-level badminton group comprised 33 students with a Chinese national second-class athlete designation or higher (18 first-class and 15 second-class athletes), at least four badminton training sessions per week, and at least 90 min per session. Training history ranged from 4 to 15 years. The recreational badminton group comprised 31 participants who had played badminton for at least one year and trained at least three times per week but had no professional badminton training or athlete rank. The non-badminton group comprised 32 participants with no badminton experience. Classification combined competitive credentials, training background, and participation history to distinguish high-level players, recreational participants, and those without badminton experience; athlete designation provided an indicator of competitive attainment. Training years were recorded descriptively for the high-level group, whereas at least one year of badminton experience was required for recreational participants. Members of all groups had sport-related educational backgrounds. Participant characteristics are summarized in Table 1. Recruitment to the non-badminton group excluded participants involved in other open-skill sports. Specific other sports, training frequency, session duration, and competitive level were not recorded, so other sport participation could not be characterized in detail.

**Table 1:** Participant characteristics.

Characteristic	Category	High-level (*n* = 33)	Recreational (*n* = 31)	Non-badminton (*n* = 32)
Age, years	Mean ± SD	19.88 ± 1.29	20.10 ± 1.14	21.06 ± 1.39
Sex	Men	20	19	24
	Women	13	12	8
Fluid intelligence	Mean ± SD	48.36 ± 6.96	48.94 ± 7.03	51.44 ± 5.70
Athlete rank	National first class	18	0	0
	National second class	15	0	0
	No badminton rank	0	31	32
Badminton experience, years	Mean ± SD	10.73 ± 2.48	1.81 ± 0.83 (*n* = 31)	0
Handedness	Left	6	4	1
	Right	27	27	31

Age and Raven score were compared using Kruskal-Wallis tests (*p* = 0.002 and *p* = 0.063); sex distribution was compared using Pearson’s chi-square test (*p* = 0.392). Handedness follows the participant information records. Badminton-experience summaries include all 31 recreational participants.

No *a priori* sample-size calculation was performed. Recruitment of high-level players was constrained by the university badminton-team roster; the recreational and non-badminton groups were then recruited to similar sizes (33, 31 and 32 participants, respectively). An illustrative sensitivity calculation for a two-*df* group effect in a Gaussian ANCOVA with five total predictors (*N* = 96) indicated 80% power at *α* = 0.05 for approximately *f*^2^ = 0.104 (partial *R*^2^ = 0.094). This describes the approximate detectable overall group effect, not the power of every pairwise or sex-subgroup comparison.

### 2.3 Measures and executive-function tasks

#### 2.3.1 Fluid intelligence

Fluid intelligence was assessed with the 60-item Standard Progressive Matrices (Raven et al., 2000). One point was awarded for each correct response. Participants completed the test individually on a mobile device in a supervised laboratory setting without a strict time limit. The response format is shown in Supplementary Figure S1.

#### 2.3.2 Word-color matching task

The task was a word-color matching judgment derived from the Stroop paradigm (Stroop, 1935; MacLeod, 1991). It was not a classical color-naming task. Stimuli combined the Chinese words for red, yellow, blue, and green with the same four font colors. Participants pressed V when word meaning and font color matched and *N* when they did not. Sixteen practice trials were followed by 96 test trials: 24 congruent and 72 incongruent. Each of the 16 word-meaning by font-color combinations appeared six times per participant. Trial order was random. Each trial comprised a 500-ms fixation cross, a stimulus displayed until response or 2000 ms, and a 500-ms blank interval. The task procedure is shown in Figure 1.

**Figure 1:**
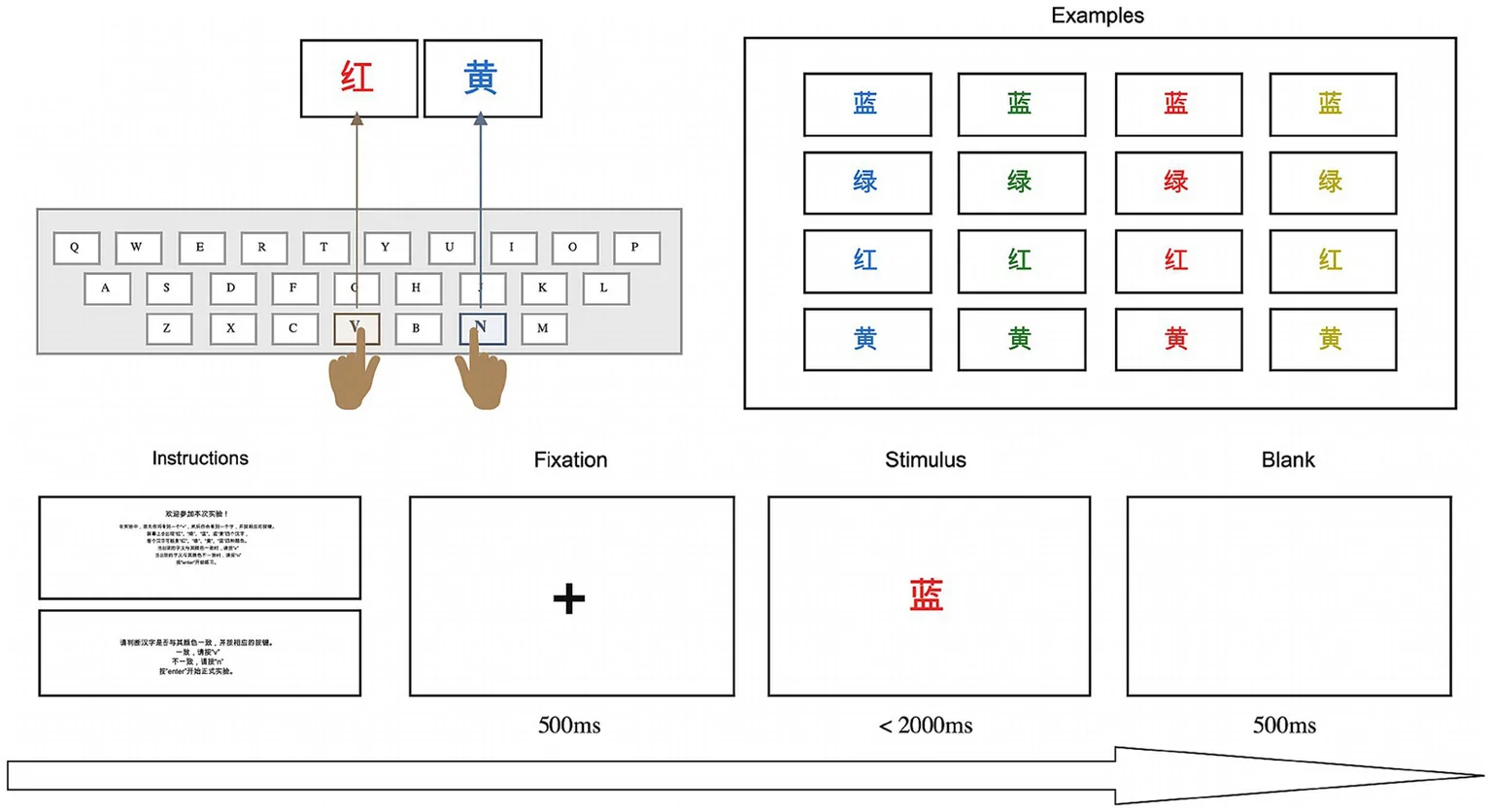
Word-color matching task schematic. The upper panels show the response-key mapping and representative stimuli. The lower panels show the instructions, fixation (500 ms), stimulus (up to 2000 ms), and blank interval (500 ms). Process labels are in English; on-screen instructions and stimuli remain in their original Chinese form.

#### 2.3.3 Task-switching paradigm

The paradigm assessed task switching (Monsell, 2003). In this implementation, the relevant judgment rule changed between color and shape. Two colored geometric figures appeared below a cue. Under the shape cue, participants ignored color and judged whether the shapes matched; under the color cue, they ignored shape and judged whether the colors matched. V indicated same and *N* indicated different. Twenty-four practice trials were followed by 144 test trials. The test comprised 18 blocks of eight trials: 48 trials in color-rule blocks, 48 in shape-rule blocks and 48 in mixed-rule blocks. Local switch and repeat outcomes were reconstructed only within the mixed blocks; excluding the first trial of each mixed block left 42 classifiable adjacent trials per participant, comprising switch and repeat trials. Across all participants, these comprised 2,152 switch and 1,880 repeat trials before response-based exclusions; both types occurred for every participant. At the start of the test, a fixation cross was displayed at the center of the screen for 4,000 ms. Stimuli were displayed until a response or the 1,500-ms response deadline. Each response or timeout was followed by a 1,500-ms blank interval before the next trial. The task procedure is shown in Figure 2.

**Figure 2:**
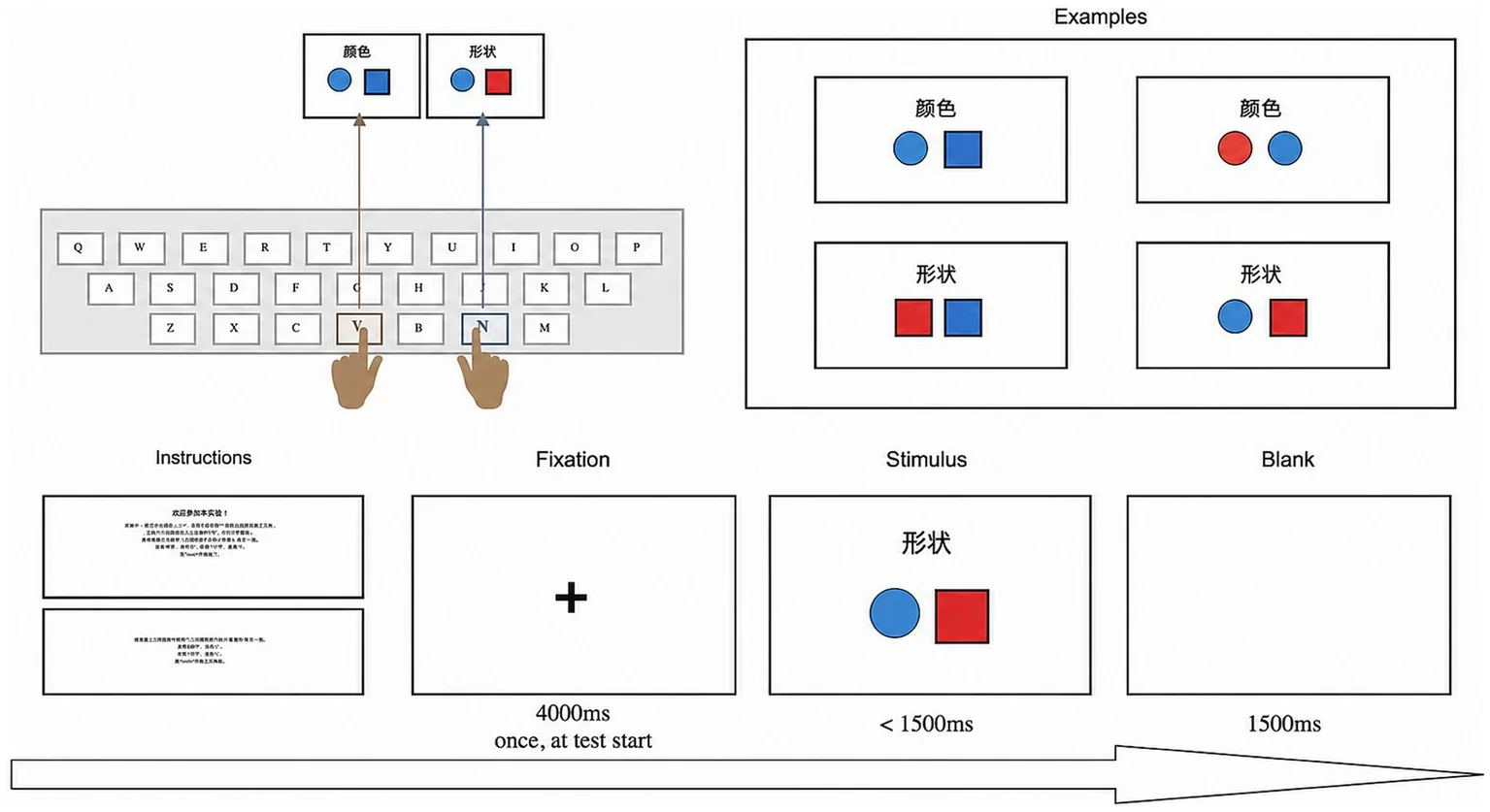
Task-switching schematic. The 4,000-ms fixation occurs once, at test start. Stimuli end after a response or 1,500 ms and are followed by a 1,500-ms blank interval. Chinese color and shape cues are retained in the schematic examples.

#### 2.3.4 1-back task

The task followed the 1-back principle introduced by Kirchner (1958). In this implementation, 26 uppercase letters were used, and participants indicated whether the current letter matched the preceding letter. *V* indicated a match and *N* a mismatch. Participants completed 22 practice trials before the formal task. The formal task comprised one initial unscored letter, followed by 20 match and 43 mismatch trials. Match trials repeated the preceding letter; mismatch trials excluded that letter and randomly selected from the remaining 25 uppercase letters. Trial counts were the same for all participants, but the specific letters could differ, and consecutive matches were allowed. Scoreable trials were presented in randomized order. Each scoreable trial included a 2000-ms blank interval and a letter presented for 500 ms. Responses not made within 500 ms were scored as incorrect. Accordingly, overall accuracy reflected both judgment accuracy and the ability to respond within the deadline. The task procedure is shown in Figure 3. The 500-ms response deadline was selected to impose time pressure in light of badminton’s rapid information-processing and response demands.

**Figure 3:**
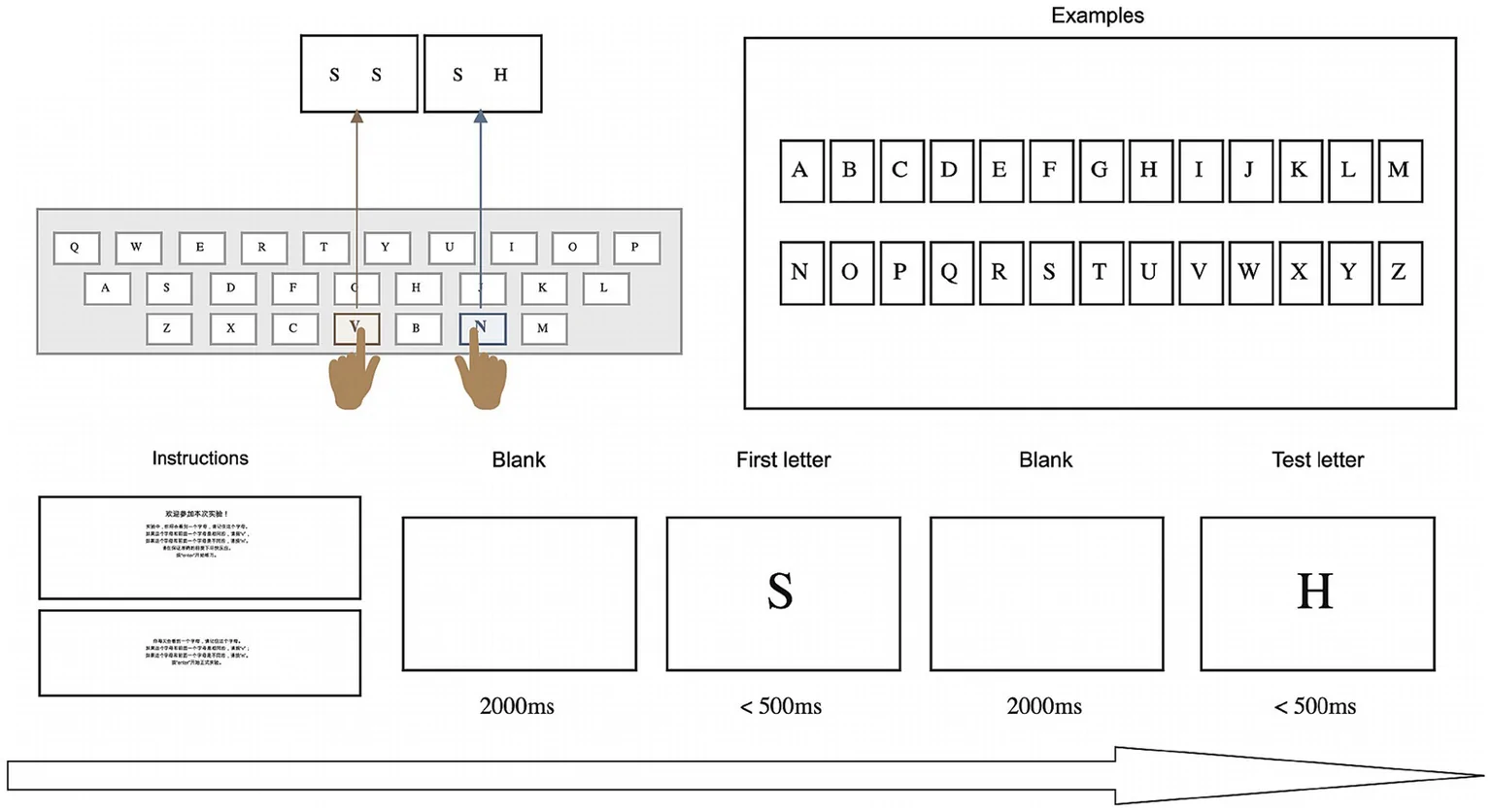
1-back task schematic. The upper panels show the response-key mapping and letter set. The lower panels show the instructions, blank interval (2000 ms), first letter (up to 500 ms), second blank interval (2000 ms), and test letter (up to 500 ms). Process labels are in English; on-screen instructions and stimuli remain in their original Chinese form.

### 2.4 Outcome construction and data processing

E-Prime 2.0 recorded trial-level responses and reaction times. Accuracy was the proportion of scoreable test trials answered correctly, including errors and timeouts in the denominator. Reaction time was calculated from correct responses with positive latency within the task deadline. During revision, task-switching labels were reconstructed within mixed blocks, excluding each block’s first trial; pure-block trials and between-block transitions were not included in local switch/repeat comparisons. The corrected mixed-block analysis contained 42 classifiable adjacent trials per participant, comprising switch and repeat trials. For 1-back, responses were scored by comparing consecutively recorded letters: V was correct for a match and *N* for a mismatch. The first letter was unscored, and accuracy was the number of correct responses divided by 63.

The revised main analyses retained all 96 participants without group-specific deletion of extreme participant-level summaries. The previously used within-group mean ± 3 SD screening was retained as a sensitivity analysis, not as the basis for the revised main sample. Corrected screened multivariate analyses included 92 participants for word-color matching, 94 for task switching, and 95 for 1-back. Supplementary Table 3 compares the screened and all-participant Gaussian analyses; the screened task-switching repeat-RT result was sensitive to this choice.

The inverse efficiency score (IES) was calculated as mean correct reaction time expressed in seconds divided by accuracy (Townsend and Ashby, 1983). Lower values indicate faster performance relative to accuracy; IES was therefore expressed in seconds. Because IES is a deterministic function of reaction time and accuracy, it was analyzed separately rather than entered into the same multivariate model. IES can also be difficult to interpret when error rates are high or the speed–accuracy relation is unstable (Bruyer and Brysbaert, 2011). IES was therefore treated as a secondary derived summary.

For the word-color task, the condition RT difference was calculated as incongruent minus congruent RT; it was not assumed to measure inhibition. For task switching, switch cost was calculated as switch minus repeat RT (Monsell, 2003; Rogers and Monsell, 1995).

### 2.5 Statistical analysis

The revised analyses were performed in Python using statsmodels and NumPyro. All 96 participants were retained. Group was categorical, with sex, age and Raven score as covariates. Task-level multivariate analyses of covariance (MANCOVAs) are reported first: the word-color and task-switching models jointly included accuracy and RT in their two conditions; the 1-back model included overall accuracy and correct-response RT. Pillai’s trace was used as the relatively robust omnibus statistic. Outcome-specific models were fitted for all tasks, but under the reporting hierarchy adopted during revision after inspecting the results, primary follow-up interpretation was restricted to tasks with an omnibus group *p* < 0.05. For tasks not meeting this criterion, component estimates are displayed for completeness rather than used to claim primary between-group differences. The three task-level tests use nominal *p* values; the hierarchy is not a study-wide familywise-error correction.

Follow-up RT outcomes and RT condition contrasts were analyzed using participant-level linear models with heteroscedasticity-consistent (HC3) covariance and *t*-based intervals (residual df = 90). Adjusted pairwise differences and simultaneous 95% confidence intervals used Bonferroni adjustment for the three group comparisons within each outcome. IES was analyzed separately as a secondary derived outcome. The original within-group mean ± 3 SD screen was retained only as a sensitivity analysis (Supplementary Tables 2, 3). RT sensitivity analyses varied the minimum threshold (positive RT, 100 ms or 150 ms), inclusion of post-error trials, and raw versus log-transformed RT. Numerical RT sensitivity results are reported in Supplementary Table 10.

Accuracy comparisons used binomial mixed models of participant-condition correct counts and scoreable denominators, including errors and timeouts in the denominator. Fixed effects were group, condition, their interaction, sex, age and Raven score. During revision, following diagnostic checks of a random-intercept model, a model with correlated participant intercepts and condition slopes was selected for reporting to accommodate condition-dependent heterogeneity. The latter used Normal (0, 2) fixed-effect priors, HalfNormal (1) random-effect standard deviations and an LKJ (2) correlation prior. Age and Raven score were standardized and sex was centered. Four Markov chain Monte Carlo (MCMC) chains each used 1,500 warmup and 2000 retained iterations. Accuracy estimates were standardized over observed covariates and integrated over participant effects. Accuracy estimates are reported with unadjusted 95% Bayesian credible intervals. Sampler diagnostics, in-sample posterior predictive checks and participant-cluster generalized estimating equation (GEE) sensitivity analyses were examined. The 1-back task remains in the main task analyses, with interpretation restricted to performance under its 500-ms response deadline. Separate binomial mixed models analyzed timeouts out of all scoreable trials and correct responses out of answered trials. Unadjusted, single-covariate and sequentially adjusted all-trial accuracy models assessed changes after covariate adjustment. Task-specific performance sensitivity analyses used one-sided exact binomial tests of answered-trial accuracy against 0.50: one excluded a participant when either condition was significantly below chance (*p* < 0.05), and a stricter rule excluded a participant when either condition was not significantly above chance (*p* ≥ 0.05). These retrospective rules supplement the all-participant primary analysis (Supplementary Tables 5, 6, 7, 8, 9). Numerical MCMC diagnostics are reported in Supplementary Table 11.

MANCOVA residual diagnostics examined multivariate normality with Mardia statistics and covariance equality with Box’s M. RT-model checks examined quadratic age/Raven terms, group-by-covariate interactions, Cook’s distance and leave-one-participant-out influence. Multivariate normality departures were detected in all three tasks; Box’s M indicated covariance heterogeneity in word-color matching (*p* < 0.001), but not task switching (*p* = 0.094) or 1-back (*p* = 0.209). For the seven RT outcomes/contrasts, joint quadratic terms (*p* ≥ 0.466) and group-by-covariate terms (*p* ≥ 0.241) did not show clear departures, and leave-one-out analyses did not change the group-test significance decisions. Given these diagnostic findings, outcome interpretation combines the task-level results with the HC3 and binomial models and their sensitivity checks. Detailed diagnostics are provided in Supplementary Table 4.

For secondary Spearman correlations, performance group was ranked as 1 for non-badminton, 2 for recreational and 3 for high-level. Rank correlation does not assume equal distances between levels but does not establish a linear expertise gradient. Fifteen task-outcome correlations were adjusted using the Benjamini-Hochberg procedure. The two word-color RT regression outcomes were retained from the original data-dependent selection and designated exploratory. These models use the same sample as the primary analyses. Sex, age and Raven score entered at step 1; high-level and recreational dummy variables entered at step 2, with non-badminton as reference. Final coefficients use HC3 standard errors and *t*-based 95% confidence intervals.

Bayesian regression model comparisons supplemented the frequentist analyses of reaction time and component-specific contrasts. Model M_0_ included sex, age, and fluid intelligence; M_1_ additionally included the two group dummy variables. Bayes factors (BFs) used a Jeffreys–Zellner–Siow (JZS) mixture-of-g prior with scale *r* = √2/4 (Rouder and Morey, 2012). Prior-robustness checks used *r* = 0.25 and *r* = 0.50. BF_10_ > 1 favored M_1_, whereas BF_01_ > 1 favored M_0_. Bayes factors were interpreted as relative evidence rather than effect sizes, in line with reporting guidance (van Doorn et al., 2021).

## 3 Results

### 3.1 Descriptive results

Raw means and standard deviations for all 96 participants are shown in Table 2, with distributions in Figure 4. Descriptive background comparisons indicated an age difference (Kruskal-Wallis *p* = 0.002), but no significant group differences in Raven scores (*p* = 0.063) or sex distribution (Pearson chi-square *p* = 0.392). Subsequent models continued to adjust statistically for sex, age, and Raven score.

**Table 2:** Raw task-outcome descriptive statistics by group.

Task	Condition	Outcome	High-level	Recreational	Non-badminton
Word-color	Congruent	Accuracy, %	83.59 ± 13.78	86.16 ± 12.14	83.59 ± 15.33
		RT, ms	609.78 ± 80.25	732.64 ± 102.09	710.96 ± 117.18
		IES, s	0.760 ± 0.215	0.873 ± 0.216	0.880 ± 0.220
	Incongruent	Accuracy, %	97.73 ± 3.93	96.06 ± 14.37	98.05 ± 2.36
		RT, ms	583.42 ± 63.26	709.47 ± 107.25	670.87 ± 112.95
		IES, s	0.597 ± 0.059	0.838 ± 0.693	0.684 ± 0.115
Task switching	Switch	Accuracy, %	85.93 ± 11.69	86.57 ± 9.98	85.08 ± 11.47
		RT, ms	873.30 ± 95.93	928.39 ± 141.90	901.07 ± 102.27
		IES, s	1.038 ± 0.206	1.086 ± 0.203	1.078 ± 0.189
	Repeat	Accuracy, %	91.81 ± 7.63	91.47 ± 11.95	93.45 ± 6.55
		RT, ms	802.61 ± 91.65	850.49 ± 111.66	833.20 ± 94.99
		IES, s	0.882 ± 0.141	0.945 ± 0.154	0.895 ± 0.113
1-back	All trials	Accuracy, %	54.45 ± 19.59	56.89 ± 21.74	47.02 ± 21.48
	Correct responses	RT, ms	430.14 ± 20.81	423.66 ± 30.28	426.43 ± 33.40
	All trials	IES, s	0.969 ± 0.630	1.083 ± 1.159	1.389 ± 1.476

Values are mean ± SD for all 96 participants: 33 high-level, 31 recreational, and 32 non-badminton. Accuracy includes errors and timeouts; RT uses correct responses within the deadline. IES is RT in seconds divided by accuracy and is a derived, non-independent measure.

**Figure 4:**
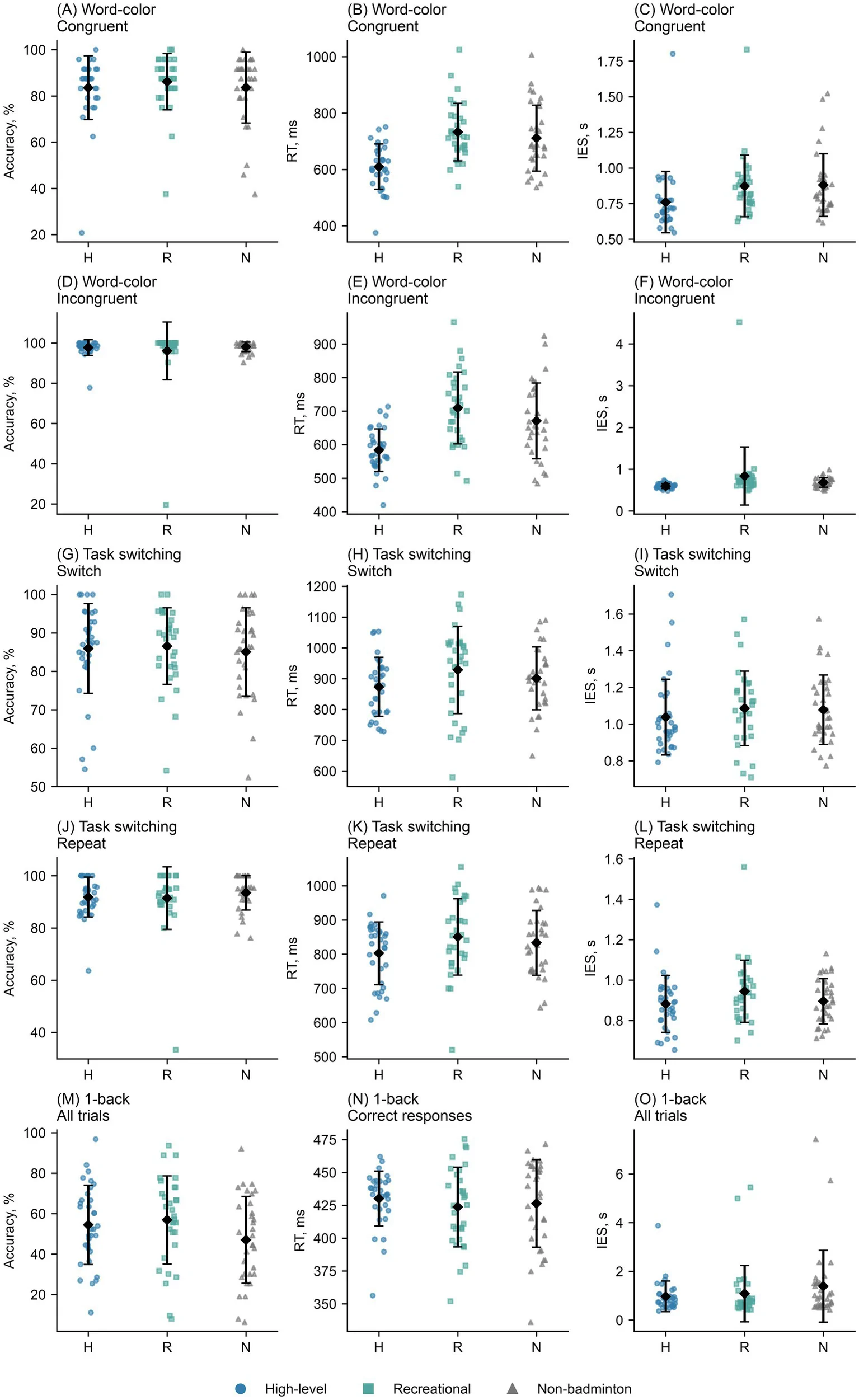
Raw distributions of executive-function task outcomes. **(A–F)** Word-color task outcomes under congruent and incongruent conditions; **(G–L)** Task-switching outcomes under switch and repeat conditions; **(M–O)** 1-back outcomes. Points show individual observations; diamonds and error bars show mean ± SD. The color key is incorporated in the figure.

### 3.2 Word-color matching task

The task-level MANCOVA showed a group effect (Pillai’s trace = 0.359, *F* (8, 176) = 4.820, *p* < 0.001). Follow-up HC3 models showed RT group differences in both conditions (congruent: *F* (2, 90) = 17.196, *p* < 0.001; incongruent: *F* (2, 90) = 17.843, *p* < 0.001). Bonferroni-adjusted comparisons showed faster responses in the high-level group than in both comparison groups, with no clear recreational versus non-badminton difference. The binomial accuracy contrasts had 95% credible intervals spanning zero. Adjusted differences and intervals are reported in Table 3.

**Table 3:** Word-color adjusted reaction-time comparisons and binomial accuracy estimates.

Outcome	Condition	Analysis	Contrast	Estimate [interval]	*p*
RT and accuracy	Task overall	MANCOVA: Pillai’s trace	All groups	*V* = 0.359; *F* (8, 176) = 4.820	<0.001
RT, ms	Congruent	HC3 group test	All groups	*F* (2, 90) = 17.196	<0.001
IES, s				*F* (2, 90) = 3.246	0.044
RT, ms	Incongruent			*F* (2, 90) = 17.843	<0.001
IES, s				*F* (2, 90) = 7.327	0.001
RT difference, ms	Incongruent—congruent			*F* (2, 90) = 1.512	0.226
RT, ms	Congruent	HC3 difference	1–2	−125.91 [−184.57, −67.24]	<0.001
			1–3	−114.47 [−179.20, −49.73]	<0.001
			2–3	11.44 [−59.00, 81.88]	1.000
	Incongruent		1–2	−127.40 [−181.66, −73.14]	<0.001
			1–3	−89.91 [−152.53, −27.28]	0.002
			2–3	37.49 [−30.41, 105.40]	0.544
Accuracy, pp	Congruent	Binomial difference	1–2	−2.52 [−8.45, 3.55]	Not applicable
			1–3	−0.00 [−6.86, 6.78]	Not applicable
			2–3	2.52 [−4.21, 9.11]	Not applicable
	Incongruent		1–2	−0.38 [−2.60, 1.69]	Not applicable
			1–3	−0.19 [−2.48, 2.05]	Not applicable
			2–3	0.19 [−1.89, 2.39]	Not applicable

All models use 96 participants and adjust for sex, age and Raven score. Groups: 1, high-level; 2, recreational; 3, non-badminton. HC3 reaction-time differences are in ms; their intervals and p values use Bonferroni adjustment for three comparisons within each outcome. Accuracy differences are percentage points (pp), with unadjusted 95% Bayesian credible intervals from a binomial model with correlated participant intercepts and condition slopes. IES is secondary.

Incongruent responses were faster than congruent responses in all three groups. The group effect on the incongruent-minus-congruent RT difference was not significant, *F* (2, 90) = 1.512, *p* = 0.226. Because condition, response hand and response frequency were confounded, this contrast cannot isolate a conflict effect or a proportion-congruency effect. The clearest between-group finding concerned response speed shared across both conditions (Figure 5).

**Figure 5:**
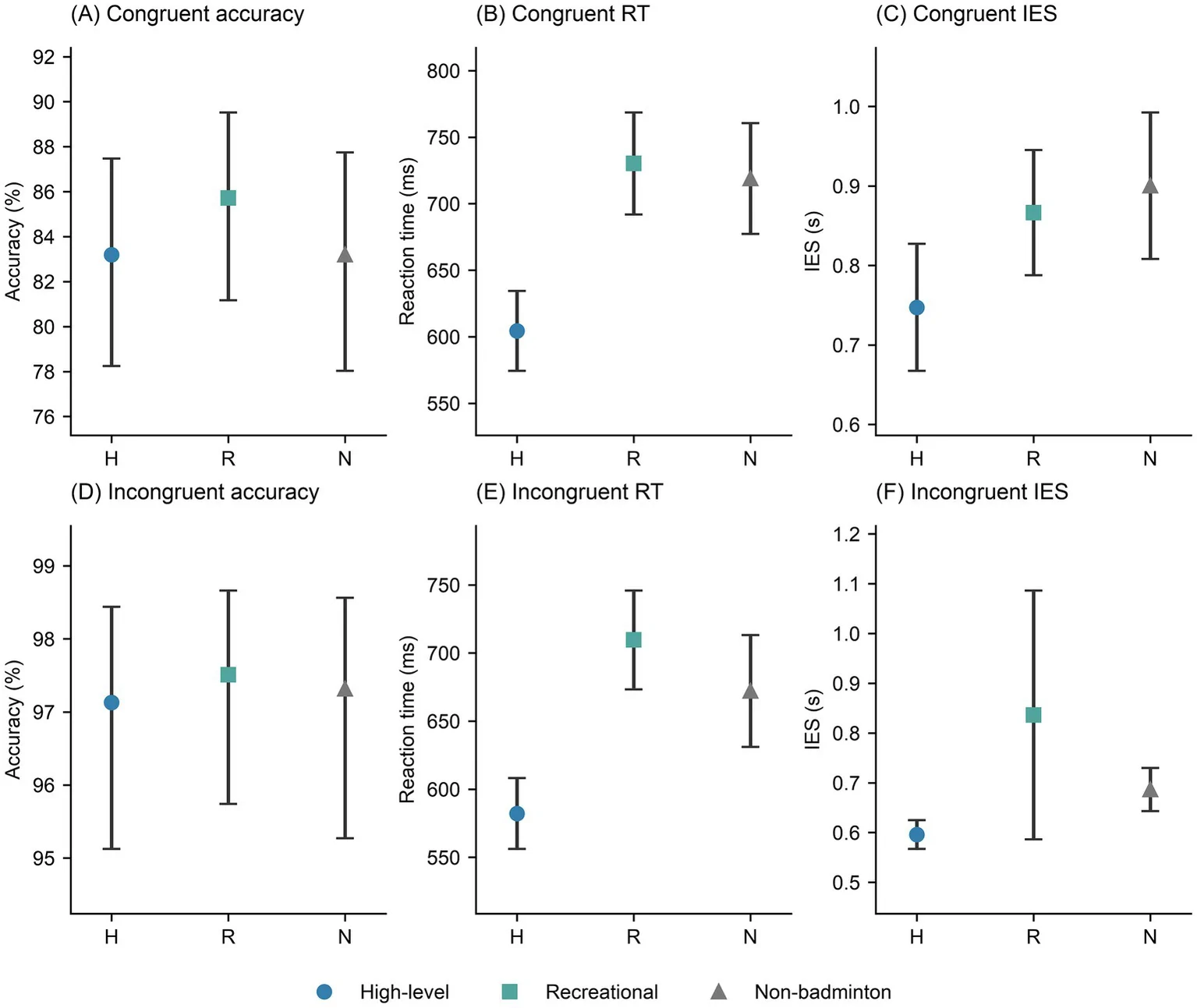
Adjusted word-color performance. **(A–C)** Congruent accuracy, RT and IES; **(D–F)** Incongruent accuracy, RT and IES. Accuracy points are population-standardized estimates from the binomial random-intercept/condition-slope model, with unadjusted 95% credible intervals. RT and IES points are covariate-adjusted linear-model means with HC3 pointwise 95% confidence intervals. Group contrasts are reported in the corresponding table. *H*, high-level; *R*, recreational; *N*, non-badminton.

The word-color RT pattern was retained across positive correct RTs, 100-ms and 150-ms minimum thresholds, exclusion of post-error trials and log-transformed RTs. Supplementary Table 10 reports all 35 models, including sample sizes, retained trial counts, *F* tests and adjusted pairwise differences.

The all-participant HC3 group tests for IES were *F* (2, 90) = 3.246, *p* = 0.044 for congruent trials and *F* (2, 90) = 7.327, *p* = 0.001 for incongruent trials. IES jointly reflects RT and accuracy and was therefore interpreted as a supporting outcome, particularly where error rates were high.

The recreational group’s incongruent IES was 0.838 s (SD = 0.693). A descriptive check omitting participant #213 gave 0.715 s (SD = 0.114; n = 30), indicating substantial influence on dispersion. The full-sample primary analysis was retained. Performance-screening sensitivity results are reported in Supplementary Tables 8, 9.

### 3.3 Task-switching paradigm

The task-level MANCOVA did not show a group effect (Pillai’s trace = 0.071, *F* (8, 176) = 0.812, *p* = 0.592). Component estimates are presented descriptively in Table 4 and Figure 6 under the revised hierarchy. Their nominal HC3 group tests were *p* = 0.225 for switch RT, *p* = 0.203 for repeat RT and *p* = 0.936 for switch cost; binomial accuracy intervals spanned zero in both conditions. These component results do not provide evidence of a task-switching advantage.

**Table 4:** Descriptive task-switching component estimates following a nonsignificant overall test.

Outcome	Condition	Analysis	Contrast	Estimate [interval]	*p*
RT and accuracy	Task overall	MANCOVA: Pillai’s trace	All groups	*V* = 0.071; *F* (8, 176) = 0.812	0.592
RT, ms	Switch	HC3 group test	All groups	*F* (2, 90) = 1.515	0.225
IES, s				*F* (2, 90) = 0.393	0.676
RT, ms	Repeat			*F* (2, 90) = 1.622	0.203
IES, s				*F* (2, 90) = 1.599	0.208
RT difference, ms	Switch—repeat			*F* (2, 90) = 0.066	0.936
RT, ms	Switch	HC3 difference	1–2	−55.21 [−134.01, 23.58]	0.272
			1–3	−29.39 [−103.11, 44.32]	1.000
			2–3	25.82 [−58.34, 109.98]	1.000
	Repeat		1–2	−47.39 [−112.33, 17.55]	0.235
			1–3	−27.76 [−98.03, 42.52]	1.000
			2–3	19.63 [−54.53, 93.80]	1.000
Accuracy, pp	Repeat	Binomial difference	1–2	0.09 [−3.88, 4.33]	Not applicable
			1–3	−1.67 [−5.56, 2.37]	Not applicable
			2–3	−1.76 [−6.07, 2.39]	Not applicable
	Switch		1–2	−0.13 [−5.31, 5.15]	Not applicable
			1–3	1.57 [−4.56, 7.59]	Not applicable
			2–3	1.70 [−4.02, 7.55]	Not applicable

All models use 96 participants and adjust for sex, age and Raven score. Groups: 1, high-level; 2, recreational; 3, non-badminton. HC3 reaction-time differences are in ms; their intervals and p values use Bonferroni adjustment for three comparisons within each outcome. Accuracy differences are percentage points (pp), with unadjusted 95% Bayesian credible intervals from a binomial model with correlated participant intercepts and condition slopes. IES is secondary. Task-switching component estimates are descriptive under the hierarchy specified in Section 2.5.

**Figure 6:**
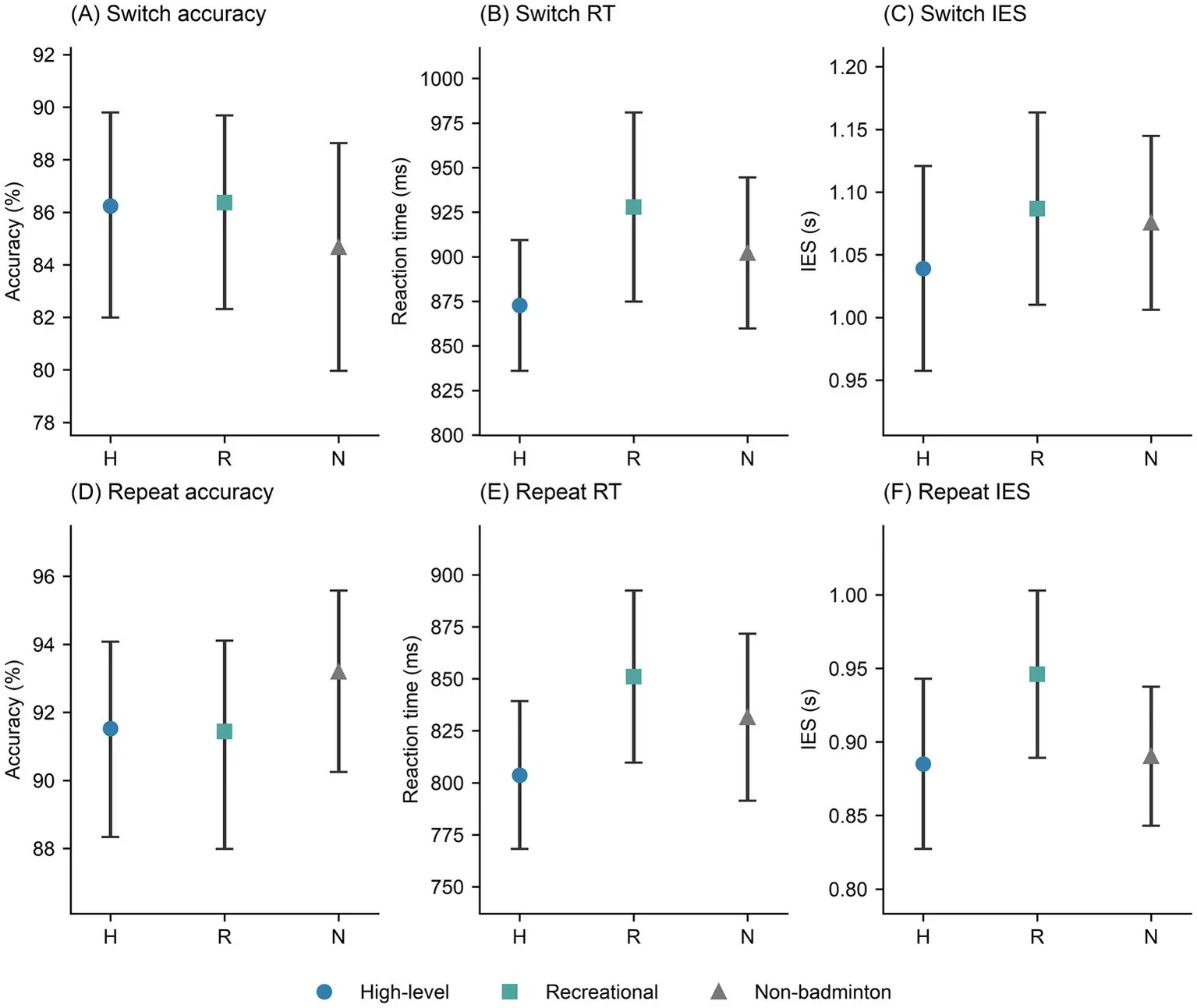
Adjusted task-switching performance. **(A–C)** Switch accuracy, RT and IES; **(D–F)** Repeat accuracy, RT and IES. Accuracy points are population-standardized estimates from the binomial random-intercept/condition-slope model, with unadjusted 95% credible intervals. RT and IES points are covariate-adjusted linear-model means with HC3 pointwise 95% confidence intervals. Group contrasts are reported in the corresponding table. *H*, high-level; *R*, recreational; *N*, non-badminton. The overall MANCOVA was nonsignificant; these component estimates are descriptive.

### 3.4 1-back task

The task-level MANCOVA showed a group effect (Pillai’s trace = 0.140, *F* (4, 180) = 3.380, *p* = 0.011). Follow-up HC3 models did not show a group difference in correct-response RT (*F* (2, 90) = 0.562, *p* = 0.572); the secondary IES test was also nonsignificant (*p* = 0.124). The binomial mixed model indicated higher deadline-constrained accuracy in both badminton groups than in the non-badminton group under match and mismatch conditions, with no clear high-level versus recreational difference (Table 5; Figure 7). These comparisons use unadjusted 95% credible intervals. GEE sensitivity results depended on specification: Holm-adjusted *p* values across three task-level joint group tests were 0.064 with exchangeable correlation and 0.0497 with working independence.

**Table 5:** 1-back adjusted reaction-time comparisons and binomial accuracy estimates.

Outcome	Condition	Analysis	Contrast	Estimate [interval]	*p*
RT and accuracy	Task overall	MANCOVA: Pillai’s trace	All groups	*V* = 0.140; *F* (4, 180) = 3.380	0.011
RT, ms	Correct responses	HC3 group test	All groups	*F* (2, 90) = 0.562	0.572
IES, s	All trials			*F* (2, 90) = 2.138	0.124
RT, ms	Correct responses	HC3 difference	1–2	5.76 [−9.97, 21.49]	1.000
			1–3	−1.73 [−21.55, 18.09]	1.000
			2–3	−7.48 [−27.88, 12.91]	1.000
Accuracy, pp	Mismatch	Binomial difference	1–2	−0.43 [−11.19, 10.19]	Not applicable
			1–3	14.29 [3.00, 25.47]	Not applicable
			2–3	14.72 [3.27, 26.03]	Not applicable
	Match		1–2	−1.95 [−11.62, 7.70]	Not applicable
			1–3	15.17 [4.01, 25.56]	Not applicable
			2–3	17.12 [6.30, 27.29]	Not applicable

All models use 96 participants and adjust for sex, age and Raven score. Groups: 1, high-level; 2, recreational; 3, non-badminton. HC3 reaction-time differences are in ms; their intervals and p values use Bonferroni adjustment for three comparisons within each outcome. Accuracy differences are percentage points (pp), with unadjusted 95% Bayesian credible intervals from a binomial model with correlated participant intercepts and condition slopes. IES is secondary. The 500-ms deadline and high timeout rate limit interpretation of the 1-back task as an updating measure. Match/mismatch status is confounded with response hand and frequency. Additional timeout and answered-trial accuracy estimates are given in Supplementary Table 6.

**Figure 7:**
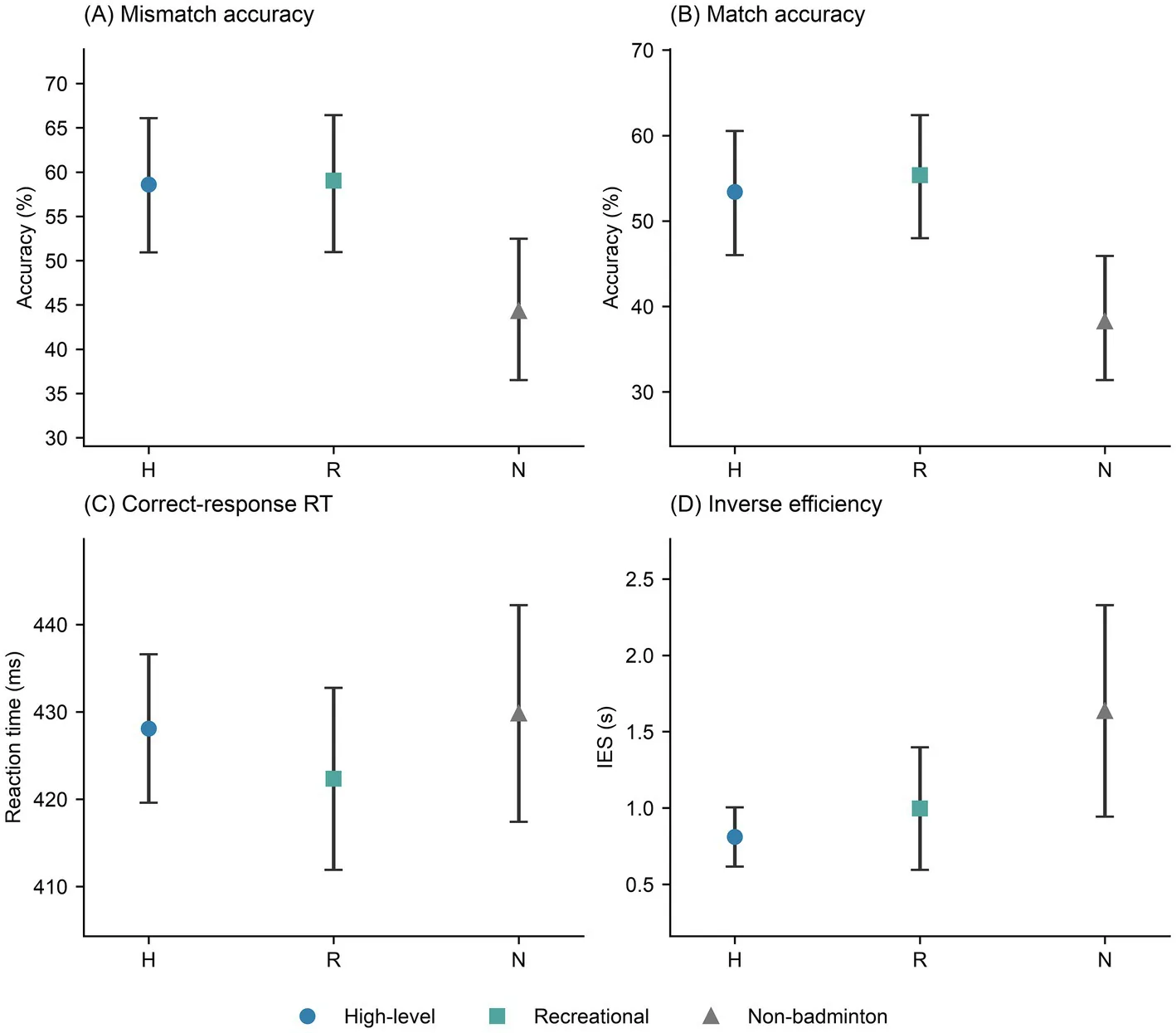
Adjusted 1-back performance under the 500-ms response deadline. **(A)** Mismatch accuracy, **(B)** Match accuracy, **(C)** Correct-response RT and **(D)** IES. Accuracy points are population-standardized estimates from the binomial random-intercept/condition-slope model, with unadjusted 95% credible intervals. RT and IES points are covariate-adjusted linear-model means with HC3 pointwise 95% confidence intervals. Group contrasts are reported in Table 5. *H*, high-level; *R*, recreational; *N*, non-badminton. All measures reflect deadline-constrained performance.

Pooled timeout rates were 40.45%, 36.92% and 48.21% in the high-level, recreational and non-badminton groups, respectively; pooled accuracy among answered trials was 91.44%, 90.18% and 90.80%. Separate covariate-adjusted binomial mixed models showed lower timeout rates in both badminton groups than in the non-badminton group under both conditions. All between-group differences in answered-trial accuracy had 95% credible intervals including zero (Supplementary Tables 5, 6). For the high-level versus non-badminton all-trial accuracy comparison, the unadjusted differences were 7.15 percentage points for mismatch trials and 7.53 for match trials, compared with 14.29 and 15.17 after full adjustment. Age adjustment contributed most to this increase, with a further contribution from Raven adjustment (Supplementary Table 7).

### 3.5 Correlation analysis

In the secondary rank correlations, word-color congruent and incongruent RT were negatively associated with badminton group rank (rho = −0.368 and −0.344; both BH-FDR *q* = 0.003). The corresponding IES correlations were rho = −0.311 (*q* = 0.008) and −0.347 (*q* = 0.003), but IES is not independent of RT and accuracy. Accuracy, task-switching outcomes and 1-back outcomes did not remain significant after correction across the 15 correlations. Supplementary Figure S2 displays the descriptive correlation matrix.

### 3.6 Hierarchical regression

For congruent reaction time, the covariate-only model explained 2.8% of variance. Adding group variables increased explained variance by 24.0%, Δ*F* (2, 90) = 14.765, *p* < 0.001. For incongruent reaction time, the covariate-only model explained 9.4%; group variables added 23.5%, Δ*F* (2, 90) = 15.771, *p* < 0.001. In the final models, the high-level group responded faster than the non-badminton group under both conditions. The recreational group did not differ from the non-badminton group. Exploratory contrasts also showed faster responses in the high-level group than in the recreational group. The pattern was therefore driven by the high-level group rather than an equally spaced performance-level gradient. Model summaries and coefficient estimates are reported in Table 6.

**Table 6:** Hierarchical regression model summaries and final-model coefficients for word-color reaction time.

A. Model summary							
Outcome	*N*	Step	*R* ^2^	Adj. *R*^2^	Δ*R*^2^	Model test	Change test
Congruent RT	96	1	0.028	−0.003	–	*F* (3, 92) = 0.891; *p* = 0.449	–
		2	0.268	0.228	0.240	*F* (5, 90) = 6.601; *p* < 0.001	Δ*F* (2, 90) = 14.765; *p* < 0.001
Incongruent RT	96	1	0.094	0.064	–	*F* (3, 92) = 3.170; *p* = 0.028	–
		2	0.329	0.292	0.235	*F* (5, 90) = 8.821; *p* < 0.001	Δ*F* (2, 90) = 15.771; *p* < 0.001

*B*. Final-model coefficients							
Outcome	Predictor	*B*	SE	*β*	*t*	*p*	95% CI
Congruent RT	High-level vs. non-badminton	−114.468	26.536	−0.482	−4.314	<0.001	[−167.19, −61.75]
	Recreational vs. non-badminton	11.438	28.875	0.047	0.396	0.693	[−45.93, 68.80]
Incongruent RT	High-level vs. non-badminton	−89.905	25.671	−0.392	−3.502	<0.001	[−140.91, −38.91]
	Recreational vs. non-badminton	37.495	27.836	0.161	1.347	0.181	[−17.81, 92.80]

Step 1 included sex, age and Raven score. Step 2 added high-level and recreational group indicators, with non-badminton as reference. B and standardized beta are OLS coefficients; SE, t tests and 95% intervals in the final-model panel use HC3 covariance. R^2^ and nested-model F tests describe the conventional OLS fits. These regressions are exploratory and use the same dataset as the primary analyses.

### 3.7 Bayesian model comparison

The group model was strongly favored for congruent and incongruent word-color RT (BF_10_ = 8574.90 and 18021.99). The condition difference yielded BF_10_ = 0.83. The corrected switch and repeat RT models yielded BF_10_ = 0.80 and 0.77, respectively, whereas switch cost favored the covariate-only model (BF_01_ = 4.73). The complementary 1-back RT model also favored the covariate-only model (BF_01_ = 3.86). These model comparisons provide complementary evidence about reaction time.

Prior-scale checks did not change these evidence directions: support remained strong for the two word-color reaction-time models, equivocal for the condition difference, and in favor of the covariate-only model for switch cost and 1-back reaction time.

## 4 Discussion

### 4.1 Word-color matching task

The central finding was faster word-color matching in the high-level group under both conditions, consistent with the response-speed prediction in H1. The binomial accuracy contrasts showed no clear group difference, and the speed pattern remained stable across the sensitivity analyses. The condition-difference component of H1 is not independently testable as a conflict effect with the present design.

Chen et al. (2018) reported shorter inhibition and change-response latencies in a change-signal task among high-level badminton players, with no statistically significant group differences in accuracy. The present matching task did not require action cancellation or restarting, so the findings should not be assigned to the same inhibitory mechanism. More broadly, athlete advantages on domain-general cognitive tasks vary by task and outcome measure (Kalén et al., 2021; Vona et al., 2024). The present pattern accords with this selective rather than uniform account.

The group difference appeared under both word-color conditions. Congruent trials required a left-hand *V* response on 25% of trials, whereas incongruent trials required a right-hand *N* response on 75%. The RT contrast therefore combines condition, response hand and response frequency and cannot be interpreted as an isolated conflict effect or proportion-congruency effect. Proportion-congruency studies distinguish stimulus–response associations from control strategies (Logan and Zbrodoff, 1979; Bugg and Crump, 2012), but the present design cannot separate these contributions. BF_10_ = 0.83 is inconclusive. The main finding is faster word-color matching in the high-level group under both conditions, which may reflect shared operations such as stimulus identification, rule matching, response selection and execution. The response-speed component of H1 was supported; its predicted conflict-related reduction in the condition contrast could not be independently evaluated.

### 4.2 Task-switching paradigm

The task-switching MANCOVA did not support H2. Although high-level players had numerically shorter RTs in both conditions, component estimates showed no clear group advantage. Bayesian comparisons for switch and repeat RT were inconclusive (BF_10_ = 0.80 and 0.77). Switch cost reflects the additional time associated with changing rules rather than overall speed; its group comparison showed no clear difference, and the supplementary Bayesian analysis favored the covariate-only model. Thus, the word-color response-speed advantage was not accompanied by evidence of a smaller switching cost in the abstract rules used here.

Koch and Krenn (2021) reported a cognitive-flexibility advantage for open-skill compared with closed-skill elite athletes, although their findings varied across flexibility measures. Their comparison concerned sport categories and included design-fluency and trail-making tests, whereas our task required switching between color and shape judgments. These abstract rule changes also differ from adapting actions to evolving play in badminton. In addition, all three groups in our study had sport-related educational backgrounds, rather than representing an athlete-versus-sedentary comparison. Task demands and comparison groups therefore provide useful context for the different findings. In the present task, an external cue specified which classification rule to apply. This places the emphasis on adapting to an explicitly instructed rule, whereas badminton involves adjusting actions as the playing situation changes. Comparing these laboratory judgments with sport-relevant switching would help clarify which aspects of flexible responding distinguish badminton performance levels.

### 4.3 1-back task

The 1-back task showed a significant overall group effect. The follow-up all-trial accuracy estimates were higher in both badminton groups than in the non-badminton group, with no clear high-level versus recreational difference or correct-response RT difference. Separate analyses clarify this pattern: both badminton groups had fewer timeouts, whereas answered-trial accuracy showed no clear group difference. The observed contrast therefore concerns timely responding under the deadline, not a progressive advantage across the three performance levels or the combined high-level accuracy and speed advantage predicted by H3.

The task required retaining the preceding letter, comparing it with the current letter and updating the reference for the next trial. The 500-ms response deadline was selected to add time pressure in relation to badminton’s rapid information-processing demands. All-trial accuracy counts timeouts as errors, whereas correct-response RT describes only successful responses completed before the deadline. Fewer timeouts can therefore accompany similar mean RT among successful responses. Fixed *V*/*N* keys also confound match/mismatch status with response hand and frequency, limiting separate interpretation of the condition contrast. The finding is best described as performance on a deadline-constrained 1-back task.

Wu et al. (2024) reported higher Corsi block span in competitive-elite than semi-elite adolescent badminton players. Their spatial-span measure, age range and performance classification differed from the present letter-based task. Here, the similar answered-trial accuracy across groups and lower timeout rates in both badminton groups emphasize timely responding rather than a demonstrated advantage in memory comparison. All-trial accuracy evidence was also sensitive to GEE specification and the stricter performance-screening rule. Varying memory load and response deadline separately would help distinguish the contributions of information retention and time available for responding.

### 4.4 Cross-task interpretation

The three tasks produced different patterns: faster word-color responses in high-level players, fewer 1-back timeouts in both badminton groups and no overall task-switching difference. Word-color RT distinguished high-level players from both comparison groups, whereas 1-back timeouts distinguished both badminton groups from participants without badminton experience. Multi-task assessment therefore identifies different aspects of performance rather than a single cognitive ranking by competitive level. Executive-function measures can reflect common and component-specific processes (Brimmell et al., 2025), and the present findings favor selective task-performance differences. Future assessments could separately measure response speed, timely responding and accuracy under different memory loads.

### 4.5 Strengths and limitations

The study compared three executive-function task paradigms within the same sample and contrasted performance levels within a single open-skill sport, thereby limiting cross-sport heterogeneity. Group comparisons were statistically adjusted for sex, age, and fluid intelligence. This adjustment does not exclude residual differences in these characteristics or confounding by unmeasured factors. Overall task outcomes were distinguished from component-specific condition contrasts, and IES was analyzed separately from its component variables.

The cross-sectional design cannot establish whether badminton participation preceded the cognitive differences. Grouping used competitive credentials, training background, and participation history, including a minimum of one year of badminton experience for recreational players. Performance level and accumulated training are closely related, so their separate contributions cannot be identified here. Although recruitment to the non-badminton group excluded other open-skill sports, detailed information on other sports, training frequency, session duration, and competitive level was not recorded. This limits comparisons of general sporting backgrounds. The sample was also not designed for sex-stratified inference. All participants completed the tasks in a fixed, non-counterbalanced order. The approximately 3-min breaks allowed rest, but practice or accumulated fatigue could still influence later performance. Testing times varied, and sleep, caffeine intake, and recent exercise were not recorded. The tasks were implemented without a separate pilot study, which limited preliminary evaluation of their timing and difficulty. Finally, the laboratory tasks do not fully reproduce badminton demands, and no independent simple or choice RT baseline was included. General response speed, motor execution, or familiarity with speeded tasks may therefore contribute to the word-color finding. Keyboard input latency and jitter were not measured against an external reference, which may affect recorded RTs and responding within the 500-ms deadline. Fixed key assignments confound condition with response hand and response frequency. Future studies could counterbalance key assignments and task order and include validated response devices, a general RT baseline and longitudinal comparisons.

## 5 Conclusion

After statistical adjustment for sex, age and fluid intelligence, high-level players responded faster in both word-color matching conditions than recreational and non-badminton participants. Both badminton groups had fewer timeouts in the deadline-constrained 1-back task, while accuracy among answered trials showed no clear group differences. All-trial 1-back accuracy evidence was sensitive to model specification. Task switching showed no overall group difference. These findings indicate task-specific performance differences rather than a broad executive-function advantage.

## Data Availability

The original contributions presented in the study are publicly available. This data can be found here: https://osf.io/u9w52/.
