# Supplementary material for: Do high-level badminton players show broad executive-function advantages? Evidence from a multi-task assessment
Source: Front Psychol. 2026 Oct 27;17:1960617. doi: 10.3389/fpsyg.2026.1960617 (PMC13747750; doi:10.3389/fpsyg.2026.1960617)
Supplement: Supplementary file 1 [file Table_1.docx]

**Supplementary Table 1.** Bayesian model comparisons across prior scales.

| **Task** | **Outcome** | ***N*** | **BF_10_** | **BF_10_ across prior scales** | **Evidence** |
| --- | --- | --- | --- | --- | --- |
| Word-color | Congruent RT | 96 | 8574.90 | 5353.00–11973.09 | *M*_1_ |
|  | Incongruent RT | 96 | 18021.99 | 12959.71–23381.54 | *M*_1_ |
|  | Condition RT difference | 96 | 0.83 | 0.64–0.99 | Equivocal |
| Task switching | Switch RT | 96 | 0.80 | 0.58–0.99 | Equivocal |
|  | Repeat RT | 96 | 0.77 | 0.56–0.95 | Equivocal |
|  | Switch cost | 96 | 0.21 | 0.13–0.31 | BF_01_ = 4.73; *M*_0_ |
| 1-back | Reaction time | 96 | 0.26 | 0.18–0.35 | BF_01_ = 3.86; *M*_0_ |

*Note. All models include 96 participants. M*_0_ *included sex, age, and fluid intelligence; M*_1_ *added two group indicators. The default prior scale was r = sqrt(2)/4; robustness checks used r = 0.25 and r = 0.50.* BF_01_ *is the reciprocal of* BF_10_*. These secondary Gaussian regression comparisons concern reaction time, not accuracy or equivalence of cognitive ability. The task-switching outcomes use corrected within-mixed-block transitions; 1-back uses response scores derived from consecutively recorded letters.*

Supplementary Table 2. Values flagged by the original within-group mean ± 3 *SD* participant-summary screen.

| **Task outcome** | **Group** | **Participant ID** | **Value** | **Flagged cells** |
| --- | --- | --- | --- | --- |
| Word-color congruent accuracy | 1 | 114 | 0.208 | 1 |
|  | 2 | 204 | 0.375 | 1 |
|  | 3 | 305 | 0.375 | 1 |
| Word-color incongruent accuracy | 1 | 114 | 0.778 | 1 |
|  | 2 | 213 | 0.194 | 1 |
|  | 3 | 305 | 0.903 | 1 |
| Task-switching switch accuracy | 2 | 206 | 0.542 | 1 |
| Task-switching repeat accuracy | 1 | 116 | 0.636 | 1 |
|  | 2 | 206 | 0.333 | 1 |
| 1-back reaction time | 1 | 129 | 356.311 | 1 |

Note. Groups: 1, high-level; 2, recreational; 3, non-badminton. The screen flagged six cells across four participants for word-color matching, three cells across two participants for task switching, and one cell in one participant for 1-back. The resulting complete-case sensitivity samples were *N* = 92, 94, and 95, respectively. The revised main analyses retain all 96 participants; this table documents the original screening rule.

Supplementary Table 3. Original screened versus all-participant Gaussian comparisons.

| **Outcome** | **All *N*** | **All *p*** | **Screened *N*** | **Screened *p*** |
| --- | --- | --- | --- | --- |
| Word-color MANCOVA (Pillai) | 96 | <0.0001 | 92 | <0.0001 |
| Task-switching MANCOVA (Pillai) | 96 | 0.5925 | 94 | 0.3821 |
| Task-switching repeat RT (ANCOVA) | 96 | 0.1759 | 94 | 0.0485 |
| 1-back MANCOVA (Pillai) | 96 | 0.0108 | 95 | 0.0105 |

Note. All-participant and screened models use the same corrected scores and covariates. Pillai's trace is the task-level omnibus statistic in the revised hierarchy; screened models are sensitivity comparisons only. Gaussian univariate RT *p* values in this table are not the HC3 *p* values reported for outcome-specific analyses. P values are nominal; the screened repeat-RT result was not stable in the full sample.

## Supplementary Table 4. Gaussian task-model and RT diagnostic checks.

| **Task** | **Mardia joint *p*** | **Box's M *p*** | **Normality assessment** |
| --- | --- | --- | --- |
| Word-color | 0.0010 | <0.001 | Departure detected |
| Task switching | 0.0010 | 0.094 | Departure detected |
| 1-back | 0.0050 | 0.209 | Departure detected |

Note. *N* = 96. Mardia *p* values use 2000 Gaussian bootstrap repetitions with the fitted design projected out and a joint skewness/kurtosis adjustment. Box's M is approximate and sensitive to nonnormality. Across the seven RT outcomes/contrasts, quadratic age/Raven tests gave *p* = 0.466-0.983 and joint group-by-covariate tests *p* = 0.241-0.995. Maximum Cook's distances ranged from 0.079 to 0.210; leave-one-participant-out group tests did not cross the 0.05 threshold relative to the full-sample decision.

Supplementary figure captions

Supplementary Figure S1. Separate examples of a Raven response screen and an eight-option item. The six-option response screen and eight-option item do not depict the same trial. Process labels are in English; on-screen instructions and test items remain in their original Chinese form.

Supplementary Figure S2. Descriptive Spearman correlation matrix. Expertise is ranked non-badminton < recreational < high-level. Outcomes 1-3: congruent word-color accuracy, RT and IES; 4-6: incongruent word-color accuracy, RT and IES; 7-9: switch accuracy, RT and IES; 10-12: repeat accuracy, RT and IES; 13-15: 1-back accuracy, RT and IES. Cells display rho, without significance symbols. The text reports BH-FDR correction for the 15 expertise-outcome correlations only.

**Supplementary Table 5. Deadline-constrained 1-back counts and observed rates.**

| Group | Condition | *N* | Trials | Answered | Timeouts | Correct | Timeout, % | Answered accuracy, % |
| --- | --- | --- | --- | --- | --- | --- | --- | --- |
| High-level | Overall | 33 | 2079 | 1238 | 841 | 1132 | 40.45 | 91.44 |
| Recreational | Overall | 31 | 1953 | 1232 | 721 | 1111 | 36.92 | 90.18 |
| Non-badminton | Overall | 32 | 2016 | 1044 | 972 | 948 | 48.21 | 90.80 |
| High-level | Mismatch | 33 | 1413 | 838 | 575 | 796 | 40.69 | 94.99 |
| Recreational | Mismatch | 31 | 1332 | 817 | 515 | 777 | 38.66 | 95.10 |
| Non-badminton | Mismatch | 32 | 1373 | 692 | 681 | 672 | 49.60 | 97.11 |
| High-level | Match | 33 | 666 | 400 | 266 | 336 | 39.94 | 84.00 |
| Recreational | Match | 31 | 621 | 415 | 206 | 334 | 33.17 | 80.48 |
| Non-badminton | Match | 32 | 643 | 352 | 291 | 276 | 45.26 | 78.41 |

Note. Rates are pooled trial counts, not averages of participant-level percentages. Answered accuracy uses answered trials as the denominator; timeouts use all scoreable trials. Counts follow the condition assignments in the analysis dataset. Each participant contributes 63 scoreable trials overall.

**Supplementary Table 6A. Adjusted 1-back timeout and answered-accuracy estimates.**

| Outcome | Condition | Group | Estimate [95% credible interval], % |
| --- | --- | --- | --- |
| Timeout | Mismatch | High-level | 38.65 [31.07, 46.87] |
|  |  | Recreational | 37.88 [30.21, 46.31] |
|  |  | Non-badminton | 53.93 [45.05, 62.44] |
|  | Match | High-level | 37.48 [30.23, 45.33] |
|  |  | Recreational | 32.10 [25.02, 39.78] |
|  |  | Non-badminton | 50.22 [41.17, 58.72] |
| Answered accuracy | Mismatch | High-level | 94.75 [92.51, 96.49] |
|  |  | Recreational | 95.31 [93.34, 96.96] |
|  |  | Non-badminton | 96.75 [94.73, 98.19] |
|  | Match | High-level | 83.30 [77.71, 87.98] |
|  |  | Recreational | 81.08 [75.17, 86.17] |
|  |  | Non-badminton | 78.29 [71.11, 84.69] |

Note. Separate binomial mixed models include group, condition, their interaction, sex, age and Raven score, with correlated participant intercepts and condition slopes. Estimates are population-standardized and intervals are unadjusted 95% Bayesian credible intervals. *N* = 96 for both models.

**Supplementary Table 6B. Adjusted 1-back timeout and answered-accuracy contrasts.**

| Outcome | Condition | Contrast | Difference [95% credible interval], pp |
| --- | --- | --- | --- |
| Timeout | Mismatch | High-level - Recreational | 0.77 [-10.10, 11.60] |
|  |  | High-level - Non-badminton | -15.28 [-26.80, -3.43] |
|  |  | Recreational - Non-badminton | -16.05 [-28.12, -3.59] |
|  | Match | High-level - Recreational | 5.37 [-4.88, 15.40] |
|  |  | High-level - Non-badminton | -12.75 [-23.84, -0.87] |
|  |  | Recreational - Non-badminton | -18.12 [-29.37, -6.15] |
| Answered accuracy | Mismatch | High-level - Recreational | -0.56 [-3.35, 2.13] |
|  |  | High-level - Non-badminton | -2.01 [-4.72, 0.75] |
|  |  | Recreational - Non-badminton | -1.45 [-3.99, 1.31] |
|  | Match | High-level - Recreational | 2.23 [-5.13, 9.59] |
|  |  | High-level - Non-badminton | 5.02 [-3.66, 14.08] |
|  |  | Recreational - Non-badminton | 2.79 [-6.01, 11.91] |

Note. pp, percentage points. Differences are first group minus second group. Credible intervals are unadjusted; they are not frequentist confidence intervals or multiplicity-adjusted tests.

**Supplementary Table 7. All-trial 1-back accuracy contrasts across covariate specifications.**

| Adjustment | Condition | Contrast | Difference [95% credible interval], pp |
| --- | --- | --- | --- |
| Unadjusted | Mismatch | High-level - Recreational | -1.40 [-12.40, 10.16] |
|  |  | High-level - Non-badminton | 7.15 [-4.33, 18.01] |
|  |  | Recreational - Non-badminton | 8.56 [-2.80, 19.83] |
|  | Match | High-level - Recreational | -3.08 [-13.73, 7.87] |
|  |  | High-level - Non-badminton | 7.53 [-2.89, 17.92] |
|  |  | Recreational - Non-badminton | 10.61 [-0.09, 21.12] |
| Sex | Mismatch | High-level - Recreational | -1.33 [-12.48, 10.04] |
|  |  | High-level - Non-badminton | 7.81 [-3.61, 19.08] |
|  |  | Recreational - Non-badminton | 9.14 [-1.99, 20.80] |
|  | Match | High-level - Recreational | -3.11 [-13.56, 7.14] |
|  |  | High-level - Non-badminton | 8.22 [-2.34, 18.83] |
|  |  | Recreational - Non-badminton | 11.33 [0.39, 21.78] |
| Age | Mismatch | High-level - Recreational | -0.69 [-11.63, 10.14] |
|  |  | High-level - Non-badminton | 11.35 [-0.40, 22.41] |
|  |  | Recreational - Non-badminton | 12.04 [0.22, 23.18] |
|  | Match | High-level - Recreational | -2.44 [-12.30, 8.19] |
|  |  | High-level - Non-badminton | 11.96 [1.19, 22.39] |
|  |  | Recreational - Non-badminton | 14.40 [3.54, 24.64] |
| Raven | Mismatch | High-level - Recreational | -1.28 [-12.08, 9.58] |
|  |  | High-level - Non-badminton | 8.85 [-2.44, 19.92] |
|  |  | Recreational - Non-badminton | 10.12 [-1.31, 21.30] |
|  | Match | High-level - Recreational | -2.93 [-13.15, 7.45] |
|  |  | High-level - Non-badminton | 9.42 [-1.00, 19.47] |
|  |  | Recreational - Non-badminton | 12.34 [1.48, 22.60] |
| Sex + age | Mismatch | High-level - Recreational | -0.49 [-11.35, 10.62] |
|  |  | High-level - Non-badminton | 11.98 [1.31, 23.11] |
|  |  | Recreational - Non-badminton | 12.48 [1.05, 23.82] |
|  | Match | High-level - Recreational | -2.22 [-12.49, 7.90] |
|  |  | High-level - Non-badminton | 12.62 [2.28, 22.83] |
|  |  | Recreational - Non-badminton | 14.84 [4.48, 25.12] |
| Sex + age + Raven | Mismatch | High-level - Recreational | -0.43 [-11.19, 10.19] |
|  |  | High-level - Non-badminton | 14.29 [3.00, 25.47] |
|  |  | Recreational - Non-badminton | 14.72 [3.27, 26.03] |
|  | Match | High-level - Recreational | -1.95 [-11.62, 7.70] |
|  |  | High-level - Non-badminton | 15.17 [4.01, 25.56] |
|  |  | Recreational - Non-badminton | 17.12 [6.30, 27.29] |

Note. All models retain *N* = 96, group-by-condition fixed effects and correlated participant intercepts and condition slopes. Only the listed covariates change. Full-adjustment estimates reproduce the submitted model. Intervals are unadjusted 95% Bayesian credible intervals. Differences between specifications describe adjustment sensitivity, not causal effects of the covariates.

**Supplementary Table 8. Performance-screening sensitivity: task and RT group tests.**

| Task | Rule | Outcome | *N* | Excluded IDs | *F* (df1, df2) | *p* |
| --- | --- | --- | --- | --- | --- | --- |
| Word-color matching | All participants | Task overall | 96 | None | 4.820 (8, 176) | <0.001 |
|  |  | Congruent RT | 96 | None | 17.196 (2, 90) | <0.001 |
|  |  | Incongruent RT | 96 | None | 17.843 (2, 90) | <0.001 |
|  |  | Condition RT contrast | 96 | None | 1.512 (2, 90) | 0.226 |
|  | Below-chance exclusion | Task overall | 95 | 114 | 4.605 (8, 174) | <0.001 |
|  |  | Congruent RT | 95 | 114 | 17.552 (2, 89) | <0.001 |
|  |  | Incongruent RT | 95 | 114 | 17.719 (2, 89) | <0.001 |
|  |  | Condition RT contrast | 95 | 114 | 1.421 (2, 89) | 0.247 |
|  | Not-above-chance exclusion | Task overall | 87 | 114,117,204,207,305,322,323,325,328 | 4.488 (8, 158) | <0.001 |
|  |  | Congruent RT | 87 | 114,117,204,207,305,322,323,325,328 | 17.239 (2, 81) | <0.001 |
|  |  | Incongruent RT | 87 | 114,117,204,207,305,322,323,325,328 | 23.562 (2, 81) | <0.001 |
|  |  | Condition RT contrast | 87 | 114,117,204,207,305,322,323,325,328 | 1.022 (2, 81) | 0.364 |
| Task switching | All participants | Task overall | 96 | None | 0.812 (8, 176) | 0.592 |
|  |  | Switch RT | 96 | None | 1.515 (2, 90) | 0.225 |
|  |  | Repeat RT | 96 | None | 1.622 (2, 90) | 0.203 |
|  |  | Switch cost | 96 | None | 0.066 (2, 90) | 0.936 |
|  | Below-chance exclusion | Task overall | 96 | None | 0.812 (8, 176) | 0.592 |
|  |  | Switch RT | 96 | None | 1.515 (2, 90) | 0.225 |
|  |  | Repeat RT | 96 | None | 1.622 (2, 90) | 0.203 |
|  |  | Switch cost | 96 | None | 0.066 (2, 90) | 0.936 |
|  | Not-above-chance exclusion | Task overall | 89 | 110,116,129,205,206,325,328 | 1.043 (8, 162) | 0.406 |
|  |  | Switch RT | 89 | 110,116,129,205,206,325,328 | 2.590 (2, 83) | 0.081 |
|  |  | Repeat RT | 89 | 110,116,129,205,206,325,328 | 1.829 (2, 83) | 0.167 |
|  |  | Switch cost | 89 | 110,116,129,205,206,325,328 | 0.790 (2, 83) | 0.457 |
| 1-back | All participants | Task overall | 96 | None | 3.380 (4, 180) | 0.011 |
|  |  | 1-back RT | 96 | None | 0.562 (2, 90) | 0.572 |
|  | Below-chance exclusion | Task overall | 95 | 317 | 3.116 (4, 178) | 0.017 |
|  |  | 1-back RT | 95 | 317 | 0.890 (2, 89) | 0.414 |
|  | Not-above-chance exclusion | Task overall | 56 | 103,104,107,109,110,117,119,122,125,126,133,204,205,207,208,209,212,214,215,216,221,223,224,228,302,304,306,310,311,314,315,316,317,318,321,323,325,326,328,332 | 2.684 (4, 100) | 0.036 |
|  |  | 1-back RT | 56 | 103,104,107,109,110,117,119,122,125,126,133,204,205,207,208,209,212,214,215,216,221,223,224,228,302,304,306,310,311,314,315,316,317,318,321,323,325,326,328,332 | 0.642 (2, 50) | 0.531 |

Note. Screening uses one-sided exact binomial tests of answered-trial accuracy against 0.50 separately by condition. Below-chance exclusion removes a participant if either condition has *p* < 0.05 for accuracy below 0.50; not-above-chance exclusion removes a participant if either condition has *p* ≥ 0.05 for accuracy above 0.50. Task overall uses MANCOVA Pillai's trace; RT group tests use HC3. Both rules are retrospective sensitivity checks; the primary analysis retains all 96 participants. P values are nominal.

**Supplementary Table 9. Performance-screening sensitivity: binomial accuracy contrasts.**

| Task | Rule | Condition | Contrast | Difference [95% credible interval], pp |
| --- | --- | --- | --- | --- |
| Word-color matching | Below-chance | Congruent | High-level - Recreational | -0.99 [-6.51, 4.38] |
|  |  |  | High-level - Non-badminton | 1.74 [-4.33, 7.89] |
|  |  |  | Recreational - Non-badminton | 2.73 [-3.22, 8.56] |
|  |  | Incongruent | High-level - Recreational | 0.01 [-1.82, 1.93] |
|  |  |  | High-level - Non-badminton | 0.21 [-1.69, 2.22] |
|  |  |  | Recreational - Non-badminton | 0.20 [-1.75, 2.26] |
|  | Not-above-chance | Congruent | High-level - Recreational | -2.00 [-5.96, 1.88] |
|  |  |  | High-level - Non-badminton | -1.68 [-6.13, 2.70] |
|  |  |  | Recreational - Non-badminton | 0.32 [-3.63, 4.53] |
|  |  | Incongruent | High-level - Recreational | 0.29 [-1.55, 2.19] |
|  |  |  | High-level - Non-badminton | -0.17 [-1.91, 1.64] |
|  |  |  | Recreational - Non-badminton | -0.46 [-2.46, 1.49] |
| Task switching | Not-above-chance | Repeat | High-level - Recreational | -0.54 [-3.85, 2.74] |
|  |  |  | High-level - Non-badminton | -0.62 [-4.12, 3.05] |
|  |  |  | Recreational - Non-badminton | -0.08 [-3.51, 3.35] |
|  |  | Switch | High-level - Recreational | 0.80 [-3.35, 4.97] |
|  |  |  | High-level - Non-badminton | 3.43 [-1.20, 8.36] |
|  |  |  | Recreational - Non-badminton | 2.62 [-2.06, 7.61] |
| 1-back | Below-chance | Mismatch | High-level - Recreational | -0.23 [-10.45, 10.48] |
|  |  |  | High-level - Non-badminton | 14.91 [3.29, 25.89] |
|  |  |  | Recreational - Non-badminton | 15.14 [3.22, 26.10] |
|  |  | Match | High-level - Recreational | -1.74 [-11.67, 8.03] |
|  |  |  | High-level - Non-badminton | 14.50 [3.43, 24.76] |
|  |  |  | Recreational - Non-badminton | 16.24 [5.43, 26.63] |
|  | Not-above-chance | Mismatch | High-level - Recreational | -5.75 [-17.42, 5.31] |
|  |  |  | High-level - Non-badminton | 9.70 [-3.51, 22.60] |
|  |  |  | Recreational - Non-badminton | 15.45 [1.95, 28.19] |
|  |  | Match | High-level - Recreational | -4.82 [-14.80, 5.41] |
|  |  |  | High-level - Non-badminton | 9.93 [-2.52, 21.56] |
|  |  |  | Recreational - Non-badminton | 14.75 [2.30, 26.52] |

Note. Rules and retained samples are reported in Supplementary Table 8. Models adjust for sex, age and Raven score and retain correlated participant intercepts and condition slopes. Differences are first group minus second group, with unadjusted 95% Bayesian credible intervals. A rule that excludes no participants reproduces the submitted primary model and is not refitted.

**Supplementary Table 10. Numerical RT sensitivity analyses.**

**A. Word-color matching: congruent**

| **Setting** | ***N*** | **Retained task trials** | ***F* (df1, df2)** | ***p*** | **High-level - recreational**  **Difference [95% CI]; *p*** | **High-level - non-badminton**  **Difference [95% CI]; *p*** | **Recreational - non-badminton**  **Difference [95% CI]; *p*** |
| --- | --- | --- | --- | --- | --- | --- | --- |
| Positive correct RT | 96 | 8670 | 17.196 (2, 90) | < .001 | -125.9  [-184.6, -67.2]  *p* < .001 | -114.5  [-179.2, -49.7]  *p* < .001 | 11.4  [-59.0, 81.9]  *p* = 1.000 |
| RT ≥ 100 ms | 96 | 8670 | 17.196 (2, 90) | < .001 | -125.9  [-184.6, -67.2]  *p* < .001 | -114.5  [-179.2, -49.7]  *p* < .001 | 11.4  [-59.0, 81.9]  *p* = 1.000 |
| RT ≥ 150 ms | 96 | 8670 | 17.196 (2, 90) | < .001 | -125.9  [-184.6, -67.2]  *p* < .001 | -114.5  [-179.2, -49.7]  *p* < .001 | 11.4  [-59.0, 81.9]  *p* = 1.000 |
| Post-error excluded | 96 | 8216 | 17.838 (2, 90) | < .001 | -124.2  [-180.3, -68.2]  *p* < .001 | -113.1  [-177.0, -49.2]  *p* < .001 | 11.2  [-56.9, 79.2]  *p* = 1.000 |
| Natural-log RT | 96 | 8670 | 15.260 (2, 90) | < .001 | -0.179  [-0.263, -0.094]  *p* < .001 | -0.161  [-0.254, -0.068]  *p* < .001 | 0.018  [-0.073, 0.109]  *p* = 1.000 |

Differences are in milliseconds except in the natural-log RT row, where they are on the ln(ms) scale.

**B. Word-color matching: incongruent**

| **Setting** | ***N*** | **Retained task trials** | ***F* (df1, df2)** | ***p*** | **High-level - recreational**  **Difference [95% CI]; *p*** | **High-level - non-badminton**  **Difference [95% CI]; *p*** | **Recreational - non-badminton**  **Difference [95% CI]; *p*** |
| --- | --- | --- | --- | --- | --- | --- | --- |
| Positive correct RT | 96 | 8670 | 17.843 (2, 90) | < .001 | -127.4  [-181.7, -73.1]  *p* < .001 | -89.9  [-152.5, -27.3]  *p* = 0.002 | 37.5  [-30.4, 105.4]  *p* = 0.544 |
| RT ≥ 100 ms | 96 | 8670 | 17.843 (2, 90) | < .001 | -127.4  [-181.7, -73.1]  *p* < .001 | -89.9  [-152.5, -27.3]  *p* = 0.002 | 37.5  [-30.4, 105.4]  *p* = 0.544 |
| RT ≥ 150 ms | 96 | 8670 | 17.843 (2, 90) | < .001 | -127.4  [-181.7, -73.1]  *p* < .001 | -89.9  [-152.5, -27.3]  *p* = 0.002 | 37.5  [-30.4, 105.4]  *p* = 0.544 |
| Post-error excluded | 96 | 8216 | 18.015 (2, 90) | < .001 | -125.9  [-178.9, -72.9]  *p* < .001 | -87.1  [-148.7, -25.4]  *p* = 0.003 | 38.8  [-27.4, 105.1]  *p* = 0.469 |
| Natural-log RT | 96 | 8670 | 15.451 (2, 90) | < .001 | -0.181  [-0.262, -0.101]  *p* < .001 | -0.126  [-0.224, -0.028]  *p* = 0.007 | 0.055  [-0.042, 0.152]  *p* = 0.510 |

Differences are in milliseconds except in the natural-log RT row, where they are on the ln(ms) scale.

**C. Word-color matching: incongruent minus congruent**

| **Setting** | ***N*** | **Retained task trials** | ***F* (df1, df2)** | ***p*** | **High-level - recreational**  **Difference [95% CI]; *p*** | **High-level - non-badminton**  **Difference [95% CI]; *p*** | **Recreational - non-badminton**  **Difference [95% CI]; *p*** |
| --- | --- | --- | --- | --- | --- | --- | --- |
| Positive correct RT | 96 | 8670 | 1.512 (2, 90) | 0.226 | -1.5  [-32.1, 29.1]  *p* = 1.000 | 24.6  [-13.6, 62.7]  *p* = 0.359 | 26.1  [-13.1, 65.3]  *p* = 0.325 |
| RT ≥ 100 ms | 96 | 8670 | 1.512 (2, 90) | 0.226 | -1.5  [-32.1, 29.1]  *p* = 1.000 | 24.6  [-13.6, 62.7]  *p* = 0.359 | 26.1  [-13.1, 65.3]  *p* = 0.325 |
| RT ≥ 150 ms | 96 | 8670 | 1.512 (2, 90) | 0.226 | -1.5  [-32.1, 29.1]  *p* = 1.000 | 24.6  [-13.6, 62.7]  *p* = 0.359 | 26.1  [-13.1, 65.3]  *p* = 0.325 |
| Post-error excluded | 96 | 8216 | 1.785 (2, 90) | 0.174 | -1.7  [-30.6, 27.3]  *p* = 1.000 | 26.0  [-11.4, 63.5]  *p* = 0.281 | 27.7  [-10.1, 65.5]  *p* = 0.232 |
| Natural-log RT | 96 | 8670 | 1.242 (2, 90) | 0.294 | -0.002  [-0.051, 0.046]  *p* = 1.000 | 0.035  [-0.026, 0.096]  *p* = 0.499 | 0.037  [-0.024, 0.098]  *p* = 0.420 |

Differences are in milliseconds except in the natural-log RT row, where they are on the ln(ms) scale.

**D. Task switching: repetition**

| **Setting** | ***N*** | **Retained task trials** | ***F* (df1, df2)** | ***p*** | **High-level - recreational**  **Difference [95% CI]; *p*** | **High-level - non-badminton**  **Difference [95% CI]; *p*** | **Recreational - non-badminton**  **Difference [95% CI]; *p*** |
| --- | --- | --- | --- | --- | --- | --- | --- |
| Positive correct RT | 96 | 3590 | 1.622 (2, 90) | 0.203 | -47.4  [-112.3, 17.5]  *p* = 0.235 | -27.8  [-98.0, 42.5]  *p* = 1.000 | 19.6  [-54.5, 93.8]  *p* = 1.000 |
| RT ≥ 100 ms | 96 | 3590 | 1.622 (2, 90) | 0.203 | -47.4  [-112.3, 17.5]  *p* = 0.235 | -27.8  [-98.0, 42.5]  *p* = 1.000 | 19.6  [-54.5, 93.8]  *p* = 1.000 |
| RT ≥ 150 ms | 96 | 3590 | 1.622 (2, 90) | 0.203 | -47.4  [-112.3, 17.5]  *p* = 0.235 | -27.8  [-98.0, 42.5]  *p* = 1.000 | 19.6  [-54.5, 93.8]  *p* = 1.000 |
| Post-error excluded | 96 | 3220 | 1.327 (2, 90) | 0.271 | -42.9  [-107.7, 21.9]  *p* = 0.330 | -24.7  [-95.0, 45.7]  *p* = 1.000 | 18.2  [-55.4, 91.8]  *p* = 1.000 |
| Natural-log RT | 96 | 3590 | 1.300 (2, 90) | 0.277 | -0.054  [-0.138, 0.030]  *p* = 0.355 | -0.037  [-0.127, 0.053]  *p* = 0.965 | 0.017  [-0.075, 0.109]  *p* = 1.000 |

Differences are in milliseconds except in the natural-log RT row, where they are on the ln(ms) scale.

**E. Task switching: switch**

| **Setting** | ***N*** | **Retained task trials** | ***F* (df1, df2)** | ***p*** | **High-level - recreational**  **Difference [95% CI]; *p*** | **High-level - non-badminton**  **Difference [95% CI]; *p*** | **Recreational - non-badminton**  **Difference [95% CI]; *p*** |
| --- | --- | --- | --- | --- | --- | --- | --- |
| Positive correct RT | 96 | 3590 | 1.515 (2, 90) | 0.225 | -55.2  [-134.0, 23.6]  *p* = 0.272 | -29.4  [-103.1, 44.3]  *p* = 1.000 | 25.8  [-58.3, 110.0]  *p* = 1.000 |
| RT ≥ 100 ms | 96 | 3590 | 1.515 (2, 90) | 0.225 | -55.2  [-134.0, 23.6]  *p* = 0.272 | -29.4  [-103.1, 44.3]  *p* = 1.000 | 25.8  [-58.3, 110.0]  *p* = 1.000 |
| RT ≥ 150 ms | 96 | 3590 | 1.515 (2, 90) | 0.225 | -55.2  [-134.0, 23.6]  *p* = 0.272 | -29.4  [-103.1, 44.3]  *p* = 1.000 | 25.8  [-58.3, 110.0]  *p* = 1.000 |
| Post-error excluded | 95 | 3220 | 2.095 (2, 89) | 0.129 | -62.2  [-138.1, 13.6]  *p* = 0.145 | -15.7  [-95.8, 64.5]  *p* = 1.000 | 46.6  [-36.8, 129.9]  *p* = 0.530 |
| Natural-log RT | 96 | 3590 | 1.135 (2, 90) | 0.326 | -0.055  [-0.149, 0.038]  *p* = 0.462 | -0.035  [-0.121, 0.052]  *p* = 1.000 | 0.021  [-0.079, 0.121]  *p* = 1.000 |

Differences are in milliseconds except in the natural-log RT row, where they are on the ln(ms) scale.

**F. Task switching: switch minus repetition**

| **Setting** | ***N*** | **Retained task trials** | ***F* (df1, df2)** | ***p*** | **High-level - recreational**  **Difference [95% CI]; *p*** | **High-level - non-badminton**  **Difference [95% CI]; *p*** | **Recreational - non-badminton**  **Difference [95% CI]; *p*** |
| --- | --- | --- | --- | --- | --- | --- | --- |
| Positive correct RT | 96 | 3590 | 0.066 (2, 90) | 0.936 | -7.8  [-62.5, 46.9]  *p* = 1.000 | -1.6  [-52.7, 49.4]  *p* = 1.000 | 6.2  [-47.5, 59.9]  *p* = 1.000 |
| RT ≥ 100 ms | 96 | 3590 | 0.066 (2, 90) | 0.936 | -7.8  [-62.5, 46.9]  *p* = 1.000 | -1.6  [-52.7, 49.4]  *p* = 1.000 | 6.2  [-47.5, 59.9]  *p* = 1.000 |
| RT ≥ 150 ms | 96 | 3590 | 0.066 (2, 90) | 0.936 | -7.8  [-62.5, 46.9]  *p* = 1.000 | -1.6  [-52.7, 49.4]  *p* = 1.000 | 6.2  [-47.5, 59.9]  *p* = 1.000 |
| Post-error excluded | 95 | 3220 | 0.302 (2, 89) | 0.740 | -8.8  [-65.1, 47.6]  *p* = 1.000 | 9.6  [-47.0, 66.3]  *p* = 1.000 | 18.4  [-39.3, 76.1]  *p* = 1.000 |
| Natural-log RT | 96 | 3590 | 0.009 (2, 90) | 0.991 | -0.001  [-0.066, 0.063]  *p* = 1.000 | 0.002  [-0.062, 0.066]  *p* = 1.000 | 0.003  [-0.059, 0.066]  *p* = 1.000 |

Differences are in milliseconds except in the natural-log RT row, where they are on the ln(ms) scale.

**G. Deadline-constrained 1-back: overall RT**

| **Setting** | ***N*** | **Retained task trials** | ***F* (df1, df2)** | ***p*** | **High-level - recreational**  **Difference [95% CI]; *p*** | **High-level - non-badminton**  **Difference [95% CI]; *p*** | **Recreational - non-badminton**  **Difference [95% CI]; *p*** |
| --- | --- | --- | --- | --- | --- | --- | --- |
| Positive correct RT | 96 | 3191 | 0.562 (2, 90) | 0.572 | 5.8  [-10.0, 21.5]  *p* = 1.000 | -1.7  [-21.5, 18.1]  *p* = 1.000 | -7.5  [-27.9, 12.9]  *p* = 1.000 |
| RT ≥ 100 ms | 96 | 3188 | 0.572 (2, 90) | 0.566 | 5.9  [-9.7, 21.4]  *p* = 1.000 | -1.4  [-21.3, 18.5]  *p* = 1.000 | -7.2  [-27.5, 13.0]  *p* = 1.000 |
| RT ≥ 150 ms | 96 | 3188 | 0.572 (2, 90) | 0.566 | 5.9  [-9.7, 21.4]  *p* = 1.000 | -1.4  [-21.3, 18.5]  *p* = 1.000 | -7.2  [-27.5, 13.0]  *p* = 1.000 |
| Post-error excluded | 92 | 2088 | 0.274 (2, 86) | 0.761 | 5.0  [-13.6, 23.5]  *p* = 1.000 | 5.1  [-16.6, 26.9]  *p* = 1.000 | 0.2  [-22.3, 22.6]  *p* = 1.000 |
| Natural-log RT | 96 | 3191 | 0.522 (2, 90) | 0.595 | 0.015  [-0.026, 0.055]  *p* = 1.000 | -0.003  [-0.054, 0.048]  *p* = 1.000 | -0.018  [-0.070, 0.035]  *p* = 1.000 |

Differences are in milliseconds except in the natural-log RT row, where they are on the ln(ms) scale.

**Notes**

All settings retain correct responses with positive RT. The 100 ms and 150 ms settings additionally apply the indicated lower bound. Post-error exclusion removes correct trials immediately following an error; the natural-log setting averages log-transformed trial RT within participants.

Models adjust for sex, age and Raven score and use HC3 robust covariance. F tests evaluate the overall group effect. Pairwise differences are first group minus second group; negative values indicate faster responses in the first group.

Pairwise p values and confidence intervals use Bonferroni correction across the three group comparisons within each model. The reported intervals have a family-wise 95% confidence level. Overall F-test p values are unadjusted.

N is the number of participants contributing to the corresponding model. It can decrease after post-error exclusion when no eligible observations remain for a participant or condition. Condition contrasts are descriptive contrasts, not isolated measures of conflict or inhibitory control.

**Supplementary Table 11. MCMC sampling diagnostics for reported binomial models.**

| Model | Chains | Warmup / retained per chain | Maximum rank R-hat | Minimum bulk ESS | Minimum tail ESS | Divergences |
| --- | --- | --- | --- | --- | --- | --- |
| Submitted 1-back | 4 | 1500 / 2000 | 1.0032 | 1655.9 | 2888.6 | 0 |
| Submitted Stroop | 4 | 1500 / 2000 | 1.0016 | 2923.0 | 4096.8 | 0 |
| Submitted Task-Switching | 4 | 1500 / 2000 | 1.0030 | 2033.2 | 2874.0 | 0 |
| 1 back below chance condition | 4 | 1500 / 2000 | 1.0026 | 1439.5 | 2342.9 | 0 |
| 1 back not above chance condition | 4 | 1500 / 2000 | 1.0027 | 1736.8 | 2942.9 | 0 |
| Stroop below chance condition | 4 | 1500 / 2000 | 1.0038 | 1914.0 | 3394.6 | 0 |
| Stroop not above chance condition | 4 | 1500 / 2000 | 1.0028 | 2428.9 | 3608.2 | 0 |
| Task Switching not above chance condition | 4 | 1500 / 2000 | 1.0035 | 2208.8 | 3450.9 | 0 |
| back acc age | 4 | 1500 / 2000 | 1.0065 | 1331.1 | 2488.6 | 0 |
| back acc raven | 4 | 1500 / 2000 | 1.0039 | 1411.7 | 2837.0 | 0 |
| back acc sex | 4 | 1500 / 2000 | 1.0044 | 951.0 | 1923.6 | 0 |
| back acc sex age | 4 | 1500 / 2000 | 1.0029 | 1328.4 | 2485.4 | 0 |
| back acc unadjusted | 4 | 1500 / 2000 | 1.0029 | 924.1 | 1635.9 | 0 |
| back answered full | 4 | 1500 / 2000 | 1.0032 | 1510.9 | 1370.3 | 0 |
| back timeout full | 4 | 1500 / 2000 | 1.0031 | 1166.4 | 2311.9 | 0 |

Note. Rank-normalized R-hat and bulk/tail effective sample sizes were recomputed from saved posterior draws. Maxima and minima range over sampled parameters. Divergence counts come from the original sampler audit for submitted models and saved sampler statistics for added models. All reported models have R-hat < 1.01, bulk/tail ESS > 400 and zero divergences. These diagnostics assess sampling quality, not whether a substantive group difference exists.
